# Technology-Enhanced Learning for Pressure Injury Prevention in Nursing: A Systematic Review and Meta-Analysis

**DOI:** 10.3390/nursrep16100371

**Published:** 2026-10-20

**Authors:** Aseel Mohammad Alzean, Abdulkareem S. Iblasi, Hassan Al Buraiki, Yupin Aungsuroch, I Gede Juanamasta

**Affiliations:** 1 Primary Health Care Corporation, Doha P.O. Box 26555, Qatar; aalzean@phcc.gov.qa; 2 Faculty of Nursing, Al Zaytoonah University of Jordan, Amman 11733, Jordan; 3 Nursing Department, College of Health Sciences, University of Buraimi, Al Buraimi 512, Oman; hassan.a@uob.edu.om; 4 Faculty of Nursing, Chulalongkorn University, Bangkok 10330, Thailand; 5 Nursing Program, Sekolah Tinggi Ilmu Kesehatan (STIKes) Wira Medika Bali, Denpasar 80239, Indonesia

**Keywords:** educational technology, e-learning, meta-analysis, nursing education, pressure ulcer, systematic review

## Abstract

**Background/Objectives:** Pressure injuries remain an important patient-safety concern, and their prevention depends on accurate knowledge among nursing staff. This review estimated the effect of technology-enhanced learning (TEL) on pressure injury knowledge among nurses and nursing students. **Methods:** Following PRISMA 2020, MEDLINE (via PubMed), Scopus, CINAHL, and CENTRAL were searched for studies published from 2000 to 2026 (final searches 7 October 2026). TEL was defined as instruction in which digital technology formed an integral component. Controlled studies reporting continuous knowledge scores were pooled with a random-effects model (Hedges’ *g*); risk of bias was assessed with RoB 2, ROBINS-I, and the NHLBI tool, and certainty of evidence with GRADE. **Results:** Five studies were included; three controlled studies (N = 277) contributed to the meta-analysis. TEL was associated with higher post-intervention knowledge than comparator conditions (*g* = 1.33, 95% CI 0.98–1.69; I^2^ = 41.5%). The effect remained significant with the Hartung–Knapp–Sidik–Jonkman adjustment (95% CI 0.55–2.12) and in leave-one-out analyses (*g* 1.17–1.49) and was smaller when an excluded physical-simulation trial was added (*g* = 1.19). Two studies synthesized narratively showed short-term gains in classification, attitudes, and knowledge, but no sustained classification advantage at three months. Certainty of evidence was low because of risk of bias and imprecision. **Conclusions:** TEL may improve short-term pressure injury knowledge among nurses and nursing students, but the small evidence base, varied comparators, and blended designs prevent attributing the effect to technology alone. Well-controlled trials measuring retention, clinical practice, and patient outcomes are needed.

## 1. Introduction

Pressure injuries remain an important patient-safety concern [1]. Evidence-based prevention requires systematic risk assessment, appropriate repositioning, skin assessment, and individualized preventive measures [2,3]. However, translating preventive recommendations into consistent clinical practice remains challenging [3]. Nurses and nursing students therefore require educational preparation that supports accurate pressure injury prevention. Although knowledge is an essential foundation for these competencies, improvements in knowledge or attitudes do not necessarily translate into reduced pressure injury incidence [4,5].

Pressure injury education commonly involves classroom teaching [3], written materials, and clinical demonstrations [6]. Educators also use simulation with standardized patients, manikins, or moulage to create realistic clinical situations [7]. Technology-enhanced learning (TEL) offers a distinct set of opportunities, delivering educational content through digital resources and interactive activities [8,9]. In this review, TEL refers to educational approaches in which digital technology forms an integral component of instruction, rather than serving solely administrative or assessment functions [8,9]. These approaches include web-based and e-learning modules, computer-based instruction, multimedia and video-based teaching, virtual simulation, serious games, and blended programmes with a substantive digital component. Physical simulation using standardized patients, manikins, task trainers, or moulage, without such a digital component, was not regarded as TEL, nor were lectures or structured packages without digital resources.

Technology-supported approaches can facilitate flexible access, repeated practice, visualization, and feedback [9]. Previous meta-analyses of e-learning in nursing education have pooled studies across many clinical topics and reported small or uncertain effects on knowledge: Lahti et al. found no statistically significant knowledge difference between e-learning and traditional learning [10], Voutilainen et al. reported test scores about five points higher on a 0–100 scale with very large heterogeneity [11], and a meta-analysis of synchronous e-learning reported a moderate effect on cognitive outcomes [12]. None of these reviews focused on pressure injury, a topic in which accurate visual recognition, classification, and risk assessment are central to prevention [4]. To our knowledge, controlled evidence on TEL for pressure injury education among nurses and nursing students has not been synthesized separately. Within pressure injury education, controlled studies have evaluated adaptive web-based learning [13], computer-based e-learning [14], blended face-to-face and video-based programmes [15], and multimedia teaching added to a digital manual [6], while other trials have compared physical simulation methods [7,16], and single-group studies have evaluated digital modules [17]. These studies differ in delivery format and comparator, which makes it important to distinguish the contribution of technology from differences in teaching intensity, content, and opportunities for practice.

The available evidence also presents methodological challenges. Studies differ in learner populations, intervention components, comparator conditions, outcome instruments, and follow-up periods. General pressure injury knowledge, classification accuracy, assessment performance, and attitudes are related but distinct educational outcomes that should not be treated as interchangeable. Furthermore, controlled studies estimate between-group differences, whereas single-group pre–post studies describe changes that cannot be attributed confidently to the intervention alone. A synthesis that distinguishes these designs and outcomes is therefore needed to support a balanced interpretation of TEL in pressure injury education.

This systematic review and meta-analysis aimed to evaluate TEL interventions addressing pressure injury prevention and assessment among nurses and nursing students. The primary objective was to estimate differences in post-intervention pressure injury knowledge between TEL interventions and concurrent comparator conditions. Secondary objectives were to describe intervention and comparator characteristics, to synthesize findings on related educational outcomes, and to assess risk of bias and the certainty of evidence.

## 2. Materials and Methods

### 2.1. Reporting and Registration

This was a systematic review of effectiveness with meta-analysis. It was conducted following the methods of the Cochrane Handbook for Systematic Reviews of Interventions [18] and is reported in accordance with the Preferred Reporting Items for Systematic Reviews and Meta-Analyses (PRISMA 2020) statement [19]. The review protocol was not prospectively registered, and no separate written protocol was published. The study was recorded in the institutional research registration system of the University of Buraimi, Oman (MSSADER); this institutional record is not a review protocol. The eligibility criteria, including the definition of TEL, were specified before the screening; changes made during the peer review (the exclusion of one physical-simulation trial on re-assessment, and the added sensitivity analyses) are identified as such.

### 2.2. Eligibility Criteria

Eligibility criteria were structured using the population, intervention, comparator, outcome, and study design (PICOS) framework. Eligibility for the systematic review was determined separately from the suitability for quantitative synthesis (Section 2.3).

*Population.* Studies enrolling pre-registration nursing students or registered nurses were eligible, in educational or clinical settings. Studies with mixed professional populations were eligible only when results for nurses or nursing students could be extracted separately. Studies conducted exclusively among informal caregivers, patient relatives, or other non-nursing groups were excluded. Nursing students and registered nurses were analysed together because both groups are being prepared for, or hold, professional responsibility for pressure injury prevention; learner population was examined as a prespecified subgroup.

*Intervention.* Eligible interventions used TEL, as defined in Section 1, to teach pressure injury prevention, assessment, or management. Eligible modalities were web-based and e-learning modules, computer-based instruction, multimedia and video-based instruction, virtual simulation, serious games, and blended programmes with digital resources formed a substantive part of instruction. Simulation was eligible only when it was delivered digitally (for example, virtual or screen-based simulation) or combined with a substantive digital component; physical simulation with standardized patients, manikins, task trainers, or moulage alone was not eligible. Interventions addressing general wound care were eligible only when pressure injury content and outcomes could be identified separately.

*Comparator.* For controlled studies, eligible comparators were conventional classroom instruction, face-to-face teaching, written materials, usual educational practice, no additional education, or an alternative educational intervention. Comparators were characterized by their instructional content and technological components. Comparisons with conventional instruction were distinguished from comparisons between a technology-supported intervention and an enhanced version of it; the latter were not interpreted as direct estimates of TEL versus non-technological education.

*Outcomes.* The primary outcome was post-intervention pressure injury knowledge, measured with a validated instrument or a clearly described structured knowledge test. Secondary outcomes were classification accuracy, assessment performance, attitudes, and self-efficacy. These were analysed separately from knowledge because they represent different constructs.

*Study designs and publications.* Randomized controlled trials, non-randomized controlled studies, and single-group pre–post studies were eligible for the review; single-group studies were eligible for narrative synthesis only. Qualitative studies, observational studies without an evaluated educational intervention, reviews, editorials, and conference abstracts without sufficient information were excluded. Peer-reviewed articles and postgraduate theses published from 2000 to 2026 with sufficient methodological and outcome information were eligible. The lower limit of 2000 was chosen because web-based and computer-mediated instruction became widely available in nursing education from that period, so earlier technologies have limited relevance to current practice. No language restriction was applied; reports required an English-language abstract and sufficient information for data extraction.

### 2.3. Eligibility for Quantitative Synthesis

Inclusion in the meta-analysis required an eligible population, a qualifying TEL intervention, a concurrent comparator group, and participant-level continuous knowledge data sufficient to calculate a between-group effect size (group sample sizes, post-intervention means, and standard deviations, or statistics from which these could be derived). Eligible studies lacking a concurrent comparator, or reporting outcomes other than continuous knowledge scores, were retained for narrative synthesis. Studies reporting only aggregated item-level response proportions were not pooled as continuous knowledge outcomes. Knowledge and classification outcomes were distinguished by what the instrument measured and how it was scored. A knowledge outcome was a participant-level score on a test of pressure injury content (facts, recognition, and decisions about prevention or management), reported as group means and standard deviations. Classification accuracy was agreement with an expert reference standard when assigning pressure injury categories to photographs. Morente et al. used an image-based questionnaire, but its 0–22 score combined six domains: wound classification, detection of infection, tissue type, peri-ulcer skin condition, dressing selection, and design of care interventions [13]. Because classification was one domain within a broader score of evaluation and management knowledge, reported per participant as means and standard deviations, this outcome was analysed as knowledge. Bredesen et al. [14] assessed only the assignment of photographs to pressure ulcer categories and Braden risk scoring, against expert ratings. Classification was reported mainly as proportions of correct photograph responses pooled across participants; a participant-level sum score was reported for one photograph set only and was compared with the Mann–Whitney U test, without means or standard deviations [14]. This outcome was therefore classified as classification accuracy, and it could not be converted to a standardized mean difference without unsupported assumptions. Because the two decisions could be questioned, the influence of Morente et al. [13] was examined in the leave-one-out analysis (Section 3.6).

### 2.4. Information Sources and Search Strategy

MEDLINE (via PubMed), CINAHL, Scopus, and the Cochrane Central Register of Controlled Trials (CENTRAL) were searched. Scopus and CINAHL were first searched between 10 and 24 January 2026 and the PubMed search was first run on 4 April 2026; the final searches of all four databases were run on 7 October 2026 (CENTRAL: Issue 10 of 12, October 2026). The final searches identified no additional eligible studies, and the record counts reported in this review are those of the final searches. Screenshots of the search histories of the final searches are provided in Supplementary Figure S4. An earlier version of this review reported searches of additional sources (Embase, Web of Science, and grey literature) whose original search records had not been retained; to ensure that the search is reproducible, it was repeated in the four databases above with full documentation, and only these searches are reported. All searches were limited to publications from 2000 to 2026; in CENTRAL this limit was applied using “Year first published”.

The strategy combined three concepts: (1) pressure ulcer or injury; (2) education, training, or simulation; and (3) nurses or nursing students. Controlled vocabulary (MeSH in PubMed; CINAHL Headings; MeSH in CENTRAL) was combined with free-text terms adapted to each database. Pressure injury terms included pressure ulcer*, pressure injur*, pressure sore*, decubitus ulcer*, and bedsore*. Educational terms included educat*, teach*, train*, learn*, simulat*, moulage, multimedia, standardized/standardised patient*, and serious game*; truncation of learn* and simulat* retrieves terms such as e-learning, online learning, and virtual simulation. The population concept used nurs*, supplemented by the MeSH headings Nurses, Students, Nursing, and Nursing Assistants in PubMed. Educational terms were deliberately broad; classification of an intervention as TEL was made during eligibility assessment rather than inferred from search terms. Terms within each concept were combined with OR and concepts with AND. The complete strategies, as recorded in each database’s search history, and the record counts are reported in Supplementary Table S1.

### 2.5. Selection Process

Records were imported into a reference-management system and duplicates were removed before screening. Two reviewers independently screened titles and abstracts and then assessed full texts against the PICOS criteria, considering the target population, pressure injury educational content, the role of technology in the intervention, and reported outcomes. Disagreements were resolved by discussion or by consultation with a third reviewer. One primary reason was recorded for each excluded full-text report, and multiple reports of the same study were linked. Eligibility for the review was determined before, and independently of, suitability for meta-analysis. During peer review, all included studies were re-assessed against the TEL criterion; one trial of physical simulation that had previously been included [16] did not meet the criterion and was excluded, and its influence is reported as a sensitivity analysis (Section 2.8).

### 2.6. Data Extraction

A standardized extraction form was developed and piloted. Two reviewers extracted study identifiers, country, design, participants, setting, sample sizes, intervention and comparator content, delivery format, duration, technological components, outcome instruments and score ranges, assessment time points, and information required for risk-of-bias assessment. For the meta-analysis, group sample sizes, post-intervention means, and standard deviations were extracted, together with baseline values where reported. When several post-intervention assessments were reported, the first assessment after completion of the intervention was used, because this time point was available in all studies, is the most comparable across them, and was chosen in advance to avoid selecting among time points according to their results. Only one knowledge outcome per study was used, so each study contributed a single, independent effect size. Each value was linked to its page and table in the source report (Supplementary Table S2). Where a report gave discrepant values (for example, between the abstract and a results table), the value from the detailed results table was used and the discrepancy recorded. Missing values were not imputed.

### 2.7. Risk of Bias Assessment

Two reviewers independently assessed risk of bias for the outcome and time point contributing to each synthesis. Randomized trials were assessed with the Cochrane RoB 2 tool (low risk, some concerns, high risk) [20], the non-randomized controlled comparison with ROBINS-I (low, moderate, serious, critical, or no information) [21], and the single-group pre–post study with the NHLBI Quality Assessment Tool for Before–After (pre–post) Studies With No Control Group (good, fair, or poor) [22]. Judgments from the different tools were not converted into a common score. Disagreements were resolved by discussion. Domain-level judgments and supporting rationale are reported in Supplementary Tables S3–S5 and Figures S1 and S2.

### 2.8. Data Synthesis and Statistical Analysis

*Effect measure.* Because studies used different knowledge instruments, effects were expressed as standardized mean differences using Hedges’ *g*, calculated from post-intervention means and the pooled standard deviation with the small-sample correction factor *J* = 1 − 3/(4[n_1_ + n_2_] − 9) [23]. The sampling variance was estimated as (n_1_ + n_2_)/(n_1_n_2_) + *g*^2^/(2[n_1_ + n_2_]). Positive values indicate higher knowledge in the TEL group.

*Model.* Effects were pooled using a random-effects model with the DerSimonian–Laird estimator of between-study variance (τ^2^) and Wald-type 95% confidence intervals [24]. Heterogeneity was quantified with Cochran’s Q, I^2^, and τ^2^ [25]. A 95% prediction interval was calculated using a *t* distribution with k − 2 degrees of freedom [26]; with three studies, there was one degree of freedom, which is reported for completeness only. Because few studies were available, the Hartung–Knapp–Sidik–Jonkman (HKSJ) adjustment was applied as a sensitivity analysis [27].

*Special designs.* For Chao et al. [6], who reported propensity-score-matched pairs without the within-pair correlation, an independent-groups variance was used; this ignores the positive within-pair correlation and is therefore likely to overestimate the variance of that study’s effect. Karimian et al. [15] reported knowledge only at three months after the intervention, which was used as the first available post-intervention assessment.

*Subgroup and sensitivity analyses.* A prespecified subgroup comparison of nursing students and registered nurses is reported descriptively, because only one study enrolled nursing students. Sensitivity analyses comprised (i) leave-one-out analysis, which also yields estimates restricted to randomized trials (omitting Chao et al. [6]) and to immediate post-intervention assessments (omitting Karimian et al. [15]); (ii) the HKSJ adjustment; (iii) the Paule–Mandel estimator of τ^2^; (iv) a common-effect (fixed-effect) model; and (v) an analysis added during peer review that included the excluded physical-simulation trial by Erdem Onder and Sari [16], first using its in situ moulage arm and then combining both moulage arms against the shared control group, as recommended in the Cochrane Handbook [18].

*Small-study effects.* A funnel plot was inspected. Formal tests for funnel-plot asymmetry were not performed because fewer than ten studies were available [28].

*Software.* Analyses were performed in Python 3.13 (Python Software Foundation, Wilmington, DE, USA; NumPy 2.5 and SciPy 1.18). Results were cross-checked against the *combine_effects* function of statsmodels 0.15 and against a Microsoft Excel spreadsheet (Microsoft Corporation, Redmond, WA, USA) that recalculates every effect size and the pooled estimate with live formulas (Supplementary Table S2, Excel file, sheet “EFFECT SIZES”). Study-level input data are given in Supplementary Table S2, study-level calculations in Supplementary Table S6, and sensitivity analyses in Supplementary Table S7. The complete analysis code is reproduced in the Supplementary Material (Code S1) and provided as a separate script.

*Narrative synthesis.* Studies and outcomes not eligible for pooling were summarized narratively, grouped by outcome domain and design, with explicit attention to design limitations and time points.

### 2.9. Certainty Assessment

The certainty of evidence for the primary outcome was assessed with the GRADE approach, considering risk of bias, inconsistency, indirectness, imprecision, and publication bias [29]. The body of evidence combined two randomized trials and one non-randomized study assessed with ROBINS-I. In line with GRADE guidance, evidence assessed with ROBINS-I may start at high certainty, with its limitations captured in the risk-of-bias judgment [30]. The body of evidence therefore started at high certainty and was rated down by one level for each serious concern; the judgment was repeated for the randomized trials alone as a check. Imprecision was judged from the confidence interval and from the optimal information size (OIS), because a large effect from a small total sample can appear precise [31]. The OIS was calculated for a two-sided α of 0.05, 80% power, and a minimal important difference of 0.3 standard deviations (a small educational effect), giving approximately 175 participants per group (350 in total). Indirectness was judged by comparing the populations, interventions, comparators, and outcome of the included studies with the review question [32]. Because the question was defined as TEL compared with any of the prespecified comparator conditions, variation among comparators was addressed by interpreting the estimate as a package effect and through sensitivity analyses. The narrower question of whether the digital component itself improves knowledge was also considered.

## 3. Results

### 3.1. Study Selection

**Figure 1:**
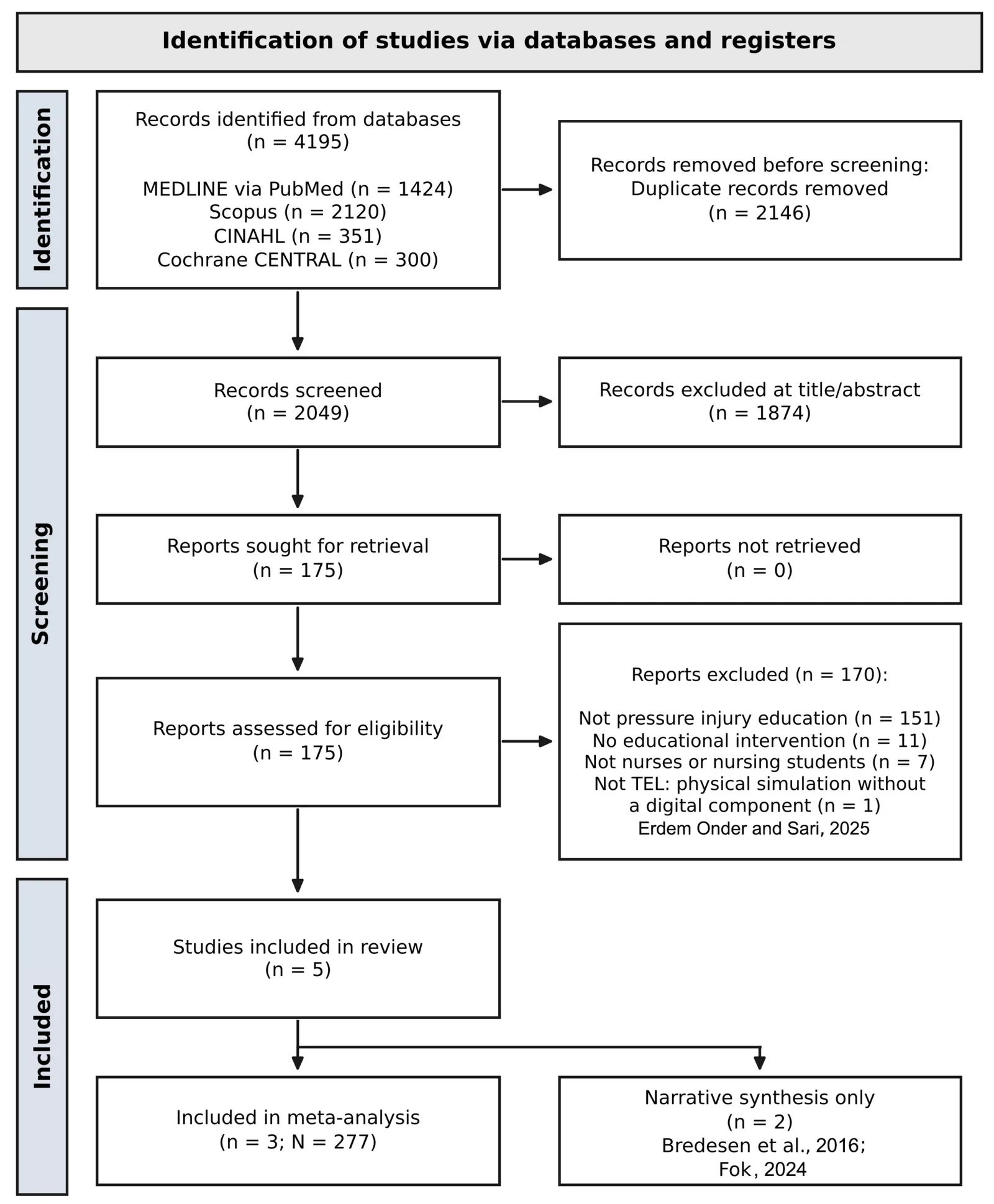
PRISMA 2020 flow diagram of study selection. Studies cited in the diagram: Bredesen et al. [14], Erdem Onder and Sari [16], and Fok [17].

The searches identified 4195 records: 1424 from MEDLINE (via PubMed), 2120 from Scopus, 351 from CINAHL, and 300 from CENTRAL. The Cochrane Library search returned 311 CENTRAL trial records (and six Cochrane Reviews, which were not exported); the publication-year limit removed 11 records published before 2000, leaving 300 records published from 2000 to 2026. After removal of 2146 duplicates, 2049 records were screened by title and abstract, and 1874 were excluded. All 175 reports sought for retrieval were obtained and assessed in full text. Of these, 170 were excluded: 151 did not address pressure injury education, 11 did not evaluate an educational intervention, 7 did not involve nurses or nursing students (including a serious-game study conducted with patient relatives), and 1 evaluated physical simulation without a digital component [16]. Five studies were included in the review, of which three contributed to the meta-analysis and two to narrative synthesis only (Figure 1). Other trials comparing physical simulation methods for pressure injury education, such as standardized patients versus manikin simulation [7], were excluded at title and abstract screening because they did not evaluate a digital intervention.

### 3.2. Study Characteristics

The five studies were published between 2014 and 2025 and conducted in Spain, Norway, Iran, Taiwan, and Canada (Table 1). Three were randomized trials [13,14,15], one was a partially randomized participant-preference trial analysed as propensity-score-matched pairs [6], and one was a single-group pre–post pilot study reported as a master’s thesis [17]. Two studies enrolled undergraduate nursing students [13,17], and three enrolled registered nurses [6,14,15].

The interventions differed in their digital components and in the contrast with the comparator (Table 1). Two studies compared a digital intervention with conventional classroom teaching of equivalent content: an adaptive web-based platform (ePULab) [13] and computer-based e-learning [14]. Karimian et al. evaluated a blended programme of face-to-face sessions and two virtual video sessions against a control group without a specified educational intervention [15]. Chao et al. compared a nursing information system (NIS) digital manual supplemented by multimedia videos, group discussion, and a workshop with the digital manual alone [6]. Fok evaluated an online module including a virtual gaming simulation focused on darker skin tones, without a comparator [17]. Thus, two of the three pooled comparisons [13,15] contrasted a TEL intervention with instruction lacking a comparable digital component, whereas the third [6] evaluated additional multimedia and face-to-face components on top of a digital manual.

Three controlled studies [6,13,15] reported continuous post-intervention knowledge scores and contributed to the meta-analysis (277 participants: 131 TEL, 146 comparator). Bredesen et al. [14] were retained for narrative synthesis because their outcome was classification performance (reported as proportions of correct photograph responses), not knowledge. Fok [17] was retained for narrative synthesis because the study had no concurrent comparator.

### 3.3. Risk of Bias

For the outcomes contributing to the meta-analysis, one randomized trial was judged to have some concerns [13], one randomized trial was judged at high risk of bias [15], and the non-randomized comparison was judged at serious risk of bias [6] (Supplementary Tables S3 and S4; Figures S1 and S2). For Morente et al. [13], allocation concealment was not reported and no prespecified analysis plan was identified. Karimian et al. [15] were judged at high risk because of unclear randomization and concealment procedures, an unspecified control condition, unreported outcome-specific attrition, self-administered outcome assessment by participants aware of their allocation, and an unexplained increase in the standard deviation of the intervention group from baseline to follow-up. For Chao et al. [6], allocation by participant availability, restriction of the analysis to matched completers (69 pairs from 218 enrolled nurses), and residual confounding despite propensity-score matching led to a judgment of serious risk.

Among the narrative studies, the immediate classification outcome in Bredesen et al. [14] was judged to have some concerns, whereas the three-month follow-up (10 and 8 participants) was at high risk because of missing outcome data. Fok’s study was rated fair on the NHLBI tool because of voluntary recruitment, a sample smaller than the calculated requirement (23 complete knowledge pairs versus 34 required), extensive survey non-completion, and a single pre- and post-measurement (Supplementary Table S5).

### 3.4. Effect of TEL on Post-Intervention Pressure Injury Knowledge

**Figure 2:**
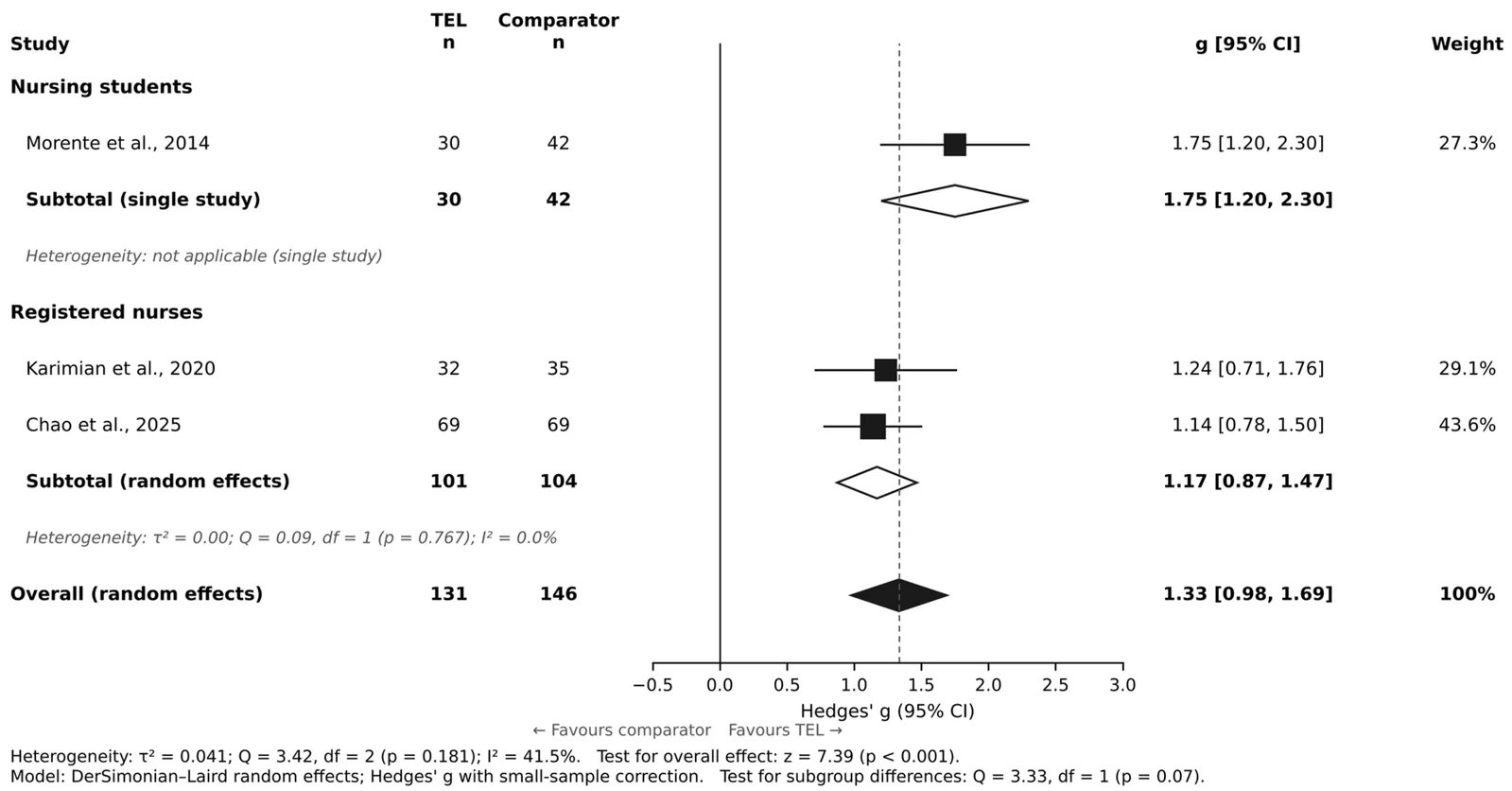
Forest plot of post-intervention pressure injury knowledge (Hedges’ *g*), by learner population. Squares are sized by random-effects weight; open diamonds show subgroup estimates, and the filled diamond the overall estimate. The dashed line marks the overall pooled effect. Studies shown: Morente et al. [13], Karimian et al. [15], and Chao et al. [6].

Individual study effects were *g* = 1.75 (95% CI 1.20–2.30) for Morente et al. [13], *g* = 1.24 (95% CI 0.71–1.76) for Karimian et al. [15], and *g* = 1.14 (95% CI 0.78–1.50) for Chao et al. [6]; all favoured the TEL group (Figure 2; Supplementary Table S6). The pooled random-effects estimate was *g* = 1.33 (95% CI 0.98–1.69; z = 7.39, *p* < 0.001), a large, standardized difference. Heterogeneity was moderate and not statistically significant (Q = 3.42, df = 2, *p* = 0.18; I^2^ = 41.5%; τ^2^ = 0.041). With only three studies, the 95% prediction interval (−2.11 to 4.78) was too wide to be informative.

The pooled estimate reflects differences between educational packages rather than a uniform comparison of TEL with non-technological instruction. In particular, both groups in Chao et al. [6] used a digital manual, and the intervention group also received face-to-face discussion and a workshop, while the intervention in Karimian et al. [15] combined face-to-face sessions with video. The findings therefore do not establish that technology alone accounted for the differences.

### 3.5. Subgroup Analysis by Learner Population

Only one study enrolled nursing students (Morente et al. [13]; 72 participants; *g* = 1.75, 95% CI 1.20–2.30). The two studies of registered nurses (Karimian et al. [15] and Chao et al. [6]; 205 participants) gave a pooled *g* of 1.17 (95% CI 0.87–1.47; I^2^ = 0%) (Figure 2). The difference between the two groups was not statistically significant (Q = 3.33, df = 1, *p* = 0.07). Because the student estimate rests on a single study whose comparison (web-based learning versus a lecture with identical slides) also differed from those in the registered-nurse studies, learner population cannot be separated from intervention and comparator differences, and this analysis is descriptive only.

### 3.6. Sensitivity Analyses

The pooled effect remained statistically significant in every sensitivity analysis (Table 2; Supplementary Table S7; Supplementary Figure S3). Leave-one-out estimates ranged from *g* = 1.17 (95% CI 0.87–1.47; I^2^ = 0%), when Morente et al. [13] was omitted, to *g* = 1.49 (95% CI 0.98–1.99), when Chao et al. [6] was omitted (randomized trials only). Omitting Karimian et al. [15] (immediate post-intervention assessments only) gave *g* = 1.41 (95% CI 0.81–2.00). The HKSJ-adjusted confidence interval was wider (0.55–2.12; *p* = 0.018); the Paule–Mandel estimator gave *g* = 1.34 (95% CI 0.98–1.69); and the common-effect estimate was *g* = 1.30 (95% CI 1.04–1.56). Adding the excluded physical-simulation trial by Erdem Onder and Sari [16] (in situ moulage arm, *g* = 0.66, 95% CI 0.12–1.20) reduced the pooled effect to *g* = 1.19 (95% CI 0.80–1.58) and increased heterogeneity (I^2^ = 61.7%); combining both of its moulage arms gave *g* = 1.12 (95% CI 0.60–1.63; I^2^ = 79.9%).

### 3.7. Small-Study Effects

**Figure 3:**
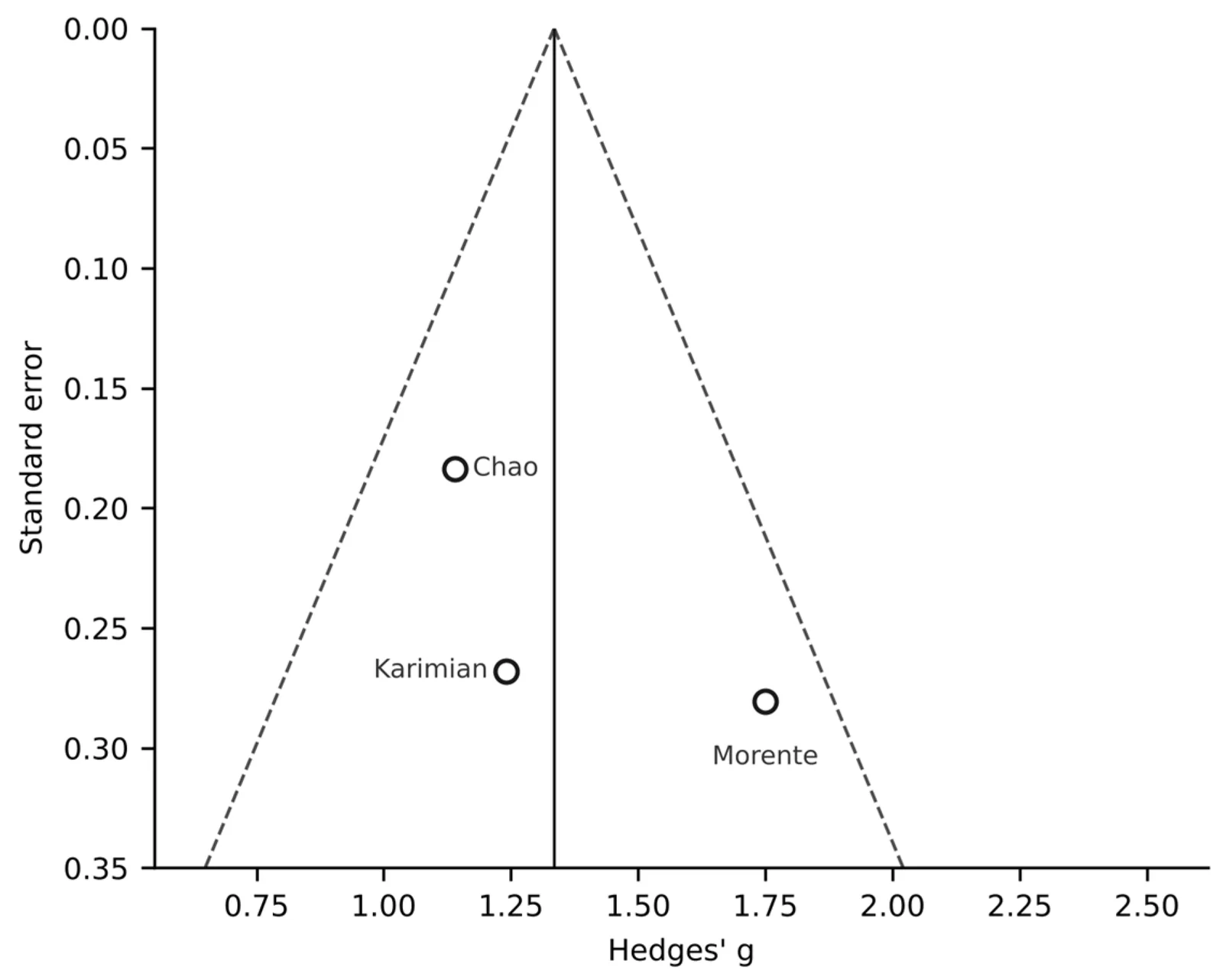
Funnel plot of the three studies contributing to the meta-analysis of post-intervention pressure injury knowledge. Dashed lines show the pseudo-95% confidence limits around the pooled estimate. Studies shown: Morente et al. [13], Karimian et al. [15], and Chao et al. [6].

The funnel plot of the three pooled studies is shown in Figure 3. With three studies, visual inspection could not establish the presence or absence of asymmetry, and no conclusion about publication bias or other small-study effects was drawn.

### 3.8. Narrative Synthesis of Other Educational Outcomes

*Classification and assessment.* Bredesen et al. [14] compared computer-based e-learning with classroom teaching of identical content among registered nurses in hospitals and nursing homes. Immediately after the intervention, the e-learning group classified a higher proportion of photographed pressure ulcers correctly (663 of 920 responses (72.1%) versus 562 of 840 (66.9%)); for photograph set A, participant sum scores differed significantly (Mann–Whitney U = 126, *p* = 0.006), whereas no clear between-group difference was observed in Braden risk-assessment accuracy. The classification advantage was not maintained at three months, but follow-up data were available for only 18 participants.

*Uncontrolled evidence.* Fok [17] evaluated an online module with a virtual gaming simulation among undergraduate nursing students. Mean knowledge scores on the Pieper–Zulkowski Pressure Injury Knowledge Test (0–72) increased from 43.57 (SD 8.90) to 53.96 (SD 10.06; *n* = 23; Wilcoxon signed-rank *p* = 0.001), attitude scores from 40.70 (SD 4.60) to 45.09 (SD 4.83; *n* = 23; *p* = 0.008), and classification scores on a ten-photograph test of darker skin tones from 4.33 (SD 1.43) to 7.17 (SD 1.63; *n* = 24; *p* < 0.001). Participants generally rated the simulation as effective. The absence of a comparator, voluntary recruitment, and survey non-completion limit attribution of these changes to the intervention, and no longer-term assessment was made.

*Attitudes and self-efficacy.* Karimian et al. [15] reported higher attitude scores in the intervention group at three months (39.37 (SD 3.21) versus 28.37 (SD 5.00); *p* < 0.001), and Chao et al. [6] reported improvements in attitudes and self-efficacy. These outcomes were not pooled with knowledge because they measure different constructs with different instruments.

Overall, the narrative evidence suggests short-term improvements in classification, attitudes, and knowledge after TEL, but it does not establish sustained retention, transfer to clinical practice, or reductions in pressure injury incidence.

### 3.9. Certainty of Evidence

Certainty of evidence for post-intervention knowledge was rated low (Table 3; Supplementary Table S9). It was downgraded by one level for risk of bias (one trial at high risk, the non-randomized comparison at serious risk, and some concerns in the remaining trial) and by one level for imprecision. Although the confidence interval excluded small effects, the 277 participants (131 and 146 per group) fell below the OIS of approximately 175 per group, and the interval widened to 0.55–2.12 with the HKSJ adjustment. Restricting the assessment to the two randomized trials (*g* = 1.49, 95% CI 0.98–1.99; 139 participants) gave the same rating: risk of bias remained serious because one trial was at high risk, and imprecision was more marked. It was not downgraded for inconsistency, because heterogeneity was moderate and all estimates favoured TEL. It was not downgraded for indirectness, because all studies enrolled the target populations, measured the primary outcome, and compared TEL with comparators within the prespecified set. However, only one study [13] compared TEL with otherwise equivalent non-digital teaching. For the narrower question of whether the digital component itself improves knowledge, the evidence would be rated down further for indirectness, to very low certainty. Publication bias could not be assessed with three studies.

## 4. Discussion

### 4.1. Principal Findings

This review included five studies of TEL for pressure injury education among nurses and nursing students. Three controlled studies (277 participants) contributed to the knowledge meta-analysis, and two were synthesized narratively. TEL interventions were associated with higher post-intervention knowledge than their comparators (*g* = 1.33, 95% CI 0.98–1.69), with moderate heterogeneity and low certainty of evidence.

The pooled result should be interpreted as a comparison of educational packages rather than an estimate of the independent effect of technology. Comparator conditions varied: one study compared web-based learning with a lecture using identical slides, one compared a blended video programme with no specified education, and one compared an enhanced digital programme with a digital manual alone. Differences in instructional content, contact time, practice opportunities, and feedback may therefore have contributed to the observed effects.

The single study of nursing students showed a larger effect than the two studies of registered nurses, but learner population was confounded with the type of comparison, and one study could not support conclusions about differences between learner groups.

The narrative evidence distinguishes immediate improvement from sustained learning. Bredesen et al. [14] reported an immediate classification benefit that was not maintained at three months, and Fok reported pre–post improvements without a comparator. Improved knowledge should not be equated with sustained changes in prevention practice or reduced pressure injury incidence.

### 4.2. Comparison with Previous Evidence

The pooled effect (*g* = 1.33) is considerably larger than those reported in broader syntheses of e-learning in nursing education. Lahti et al. found no statistically significant difference in knowledge between e-learning and traditional learning [10]; Voutilainen et al. reported scores about five points higher on a 0–100 scale, with very large heterogeneity [11]; and a meta-analysis of synchronous e-learning reported a standardized effect of 0.63 on cognitive outcomes [12]. Several features of the included studies may explain the larger effect here. First, one comparator received no specified education [15], and knowledge gains would be expected after any structured teaching. Second, knowledge was measured with instruments closely aligned with the intervention content, including a researcher-developed image-based questionnaire [13], and mostly immediately after teaching, when differences are largest. Third, the intervention group in Chao et al. [6] received additional contact time, discussion, and a workshop. Fourth, all pooled studies were small and had some or serious risk of bias, both of which are associated with inflated effect estimates. The magnitude should therefore be regarded as an upper estimate for well-resourced, content-aligned programmes rather than as the expected benefit of introducing technology into existing teaching.

Heterogeneity was moderate (I^2^ = 41.5%) and was explained largely by the larger effect in Morente et al. [13]; omitting this study reduced I^2^ to 0%. This was the study with the clearest technology-versus-conventional contrast, in which both groups received identical slide content [13], but it was also the only study of nursing students and used a researcher-developed test, so these explanations cannot be separated. Two trials comparing physical simulation methods, which fall outside the definition of TEL used here, reported a smaller difference or no difference in knowledge [7,16]; including one such trial in a sensitivity analysis reduced the pooled estimate and increased heterogeneity. This pattern is consistent with the expectation that effects depend on what the comparator already provides, and it supports keeping digital and physical simulation separate when synthesizing educational evidence.

### 4.3. Implications for Nursing Education and Clinical Practice

The findings suggest that digital educational resources may be useful components of pressure injury education for nurses and nursing students. However, the evidence does not support recommending any single digital modality as consistently superior. Educational decisions should consider the learning objective, learner characteristics, instructional content, and the alternative against which an intervention has been evaluated.

Knowledge, classification accuracy, assessment performance, attitudes, and self-efficacy should remain distinct educational targets. An intervention that improves a knowledge-test score should not be assumed to improve bedside assessment or prevention practice. For nurse educators, technology is best evaluated as part of an instructional package rather than as an isolated feature, and programme evaluation should establish whether learning gains persist and transfer to assessment and prevention activities. For clinical services, implementation should be accompanied by assessment of practice-relevant competencies rather than reliance on immediate knowledge scores alone.

### 4.4. Strengths and Limitations

This review focused specifically on pressure injury education among nurses and nursing students; applied an explicit definition of TEL; distinguished eligibility for the review from suitability for pooling; separated knowledge from other outcome domains; used design-specific risk-of-bias tools; and reports study-level data, calculations, and analysis code to allow verification. Sensitivity analyses examined the influence of individual studies, the non-randomized design, follow-up timing, the choice of estimator, and the eligibility decision for physical simulation.

Several limitations constrain the conclusions. First, the meta-analysis included only three studies with 277 participants, which limits precision and makes the heterogeneity estimate and prediction interval unreliable. Second, interventions and comparators differed, and in one pooled study both groups received digital instruction; the result is therefore not a uniform estimate of TEL versus non-technological education, and several interventions combined digital and face-to-face components. Third, the conclusions depend on the definition of TEL: excluding physical simulation was consistent with the prespecified criterion, but a previously included trial was excluded on re-assessment during peer review, and including it reduced the pooled effect to *g* = 1.19 (95% CI 0.80–1.58). Fourth, risk of bias was a concern in all pooled studies, and the matched design of Chao et al. [6] was analysed with an independent-groups variance because within-pair correlation was unavailable. Fifth, Karimian et al. [15] assessed knowledge three months after the intervention, whereas the other pooled studies assessed it immediately. Sixth, evidence on retention, clinical practice, and patient outcomes was insufficient, and the narrative studies were limited by missing follow-up data (Bredesen et al. [14]) and an uncontrolled design with incomplete data (Fok [17]). Finally, publication bias could not be assessed; the protocol was not prospectively registered; and the restriction to four databases (Embase, Web of Science, and grey-literature sources were not part of the documented search) and to publications from 2000 onwards may have missed relevant studies.

### 4.5. Recommendations for Future Research

Future trials should separate the effects of digital delivery from those of additional content, contact time, feedback, or practice, ideally by comparing interventions with equivalent content and exposure. Controlled studies should report allocation concealment, participant flow, intervention fidelity, and prespecified analysis plans, and should be prospectively registered. Matched studies should report effect estimates and variances that account for matching.

Outcome assessment should extend beyond immediate knowledge scores to include retention, classification and assessment performance, observed prevention practice, and, where feasible, patient outcomes, analysed separately with construct-appropriate instruments. Fok’s focus on darker skin tones highlights an important and under-represented educational area that warrants controlled evaluation. Future syntheses could also compare digital and physical simulation directly, as distinct educational approaches. Larger, rigorously conducted studies would allow assessment of differences by learner population, clinical setting, and intervention characteristics.

## 5. Conclusions

TEL interventions may improve short-term pressure injury knowledge among nurses and nursing students compared with their respective comparator conditions (*g* = 1.33, 95% CI 0.98–1.69; three studies; low-certainty evidence). Because the comparisons did not uniformly evaluate TEL against non-technological instruction and several interventions combined digital and face-to-face components, the observed effects cannot be attributed to technology alone. The small evidence base and the risk of bias limit confidence in the magnitude of benefit, and the improvements in knowledge should not be interpreted as evidence of sustained changes in clinical practice or reduced pressure injury incidence. Well-controlled studies should distinguish digital delivery from other instructional components and assess retention, clinical assessment performance, prevention practices, and patient outcomes.

## Figures and Tables

**Table 1:** Characteristics of the included studies.

Study (Country)	Population; Design	TEL Intervention (Digital Component)	Comparator	Outcome; Instrument; Time Point	Nature of Contrast	Synthesis
Morente et al., 2014 [13] (Spain)	Undergraduate nursing students; RCT (*n* = 30/42)	Adaptive web-based self-learning platform (ePULab); 4 h	Classroom lecture using the same slides; 4 h	Applied evaluation knowledge; image-based questionnaire (0–22); immediately post-session	TEL vs. conventional, equivalent content and time	Meta-analysis
Bredesen et al., 2016 [14] (Norway)	Registered nurses, hospitals and nursing homes; block-randomized trial (*n* = 23/21)	Computer-based e-learning on risk assessment and classification	Classroom lecture, identical content	Classification (40 photographs; proportion correct) and Braden accuracy; immediate and 3 months	TEL vs. conventional, equivalent content	Narrative: outcome is classification, not knowledge
Karimian et al., 2020 [15] (Iran)	ICU nurses; RCT (*n* = 32/35)	Blended: 4 face-to-face sessions + 2 virtual video sessions, brochures, pocket cards	Control; no specified educational intervention	Knowledge, PUKT (0–47); attitudes, APuP; 3 months post-intervention	Blended TEL vs. no specified education	Meta-analysis (knowledge); attitudes narrative
Chao et al., 2025 [6] (Taiwan)	Registered nurses, mixed settings; partially randomized participant-preference trial, propensity-score matched (69 pairs)	NIS digital manual + 4 multimedia videos (66 min), group discussion, and workshop (160 min)	NIS digital manual alone	Knowledge, PZ-PUKT (0–72); attitudes; self-efficacy; immediately post-workshop	Add-on: enhanced digital programme vs. digital manual (both arms digital)	Meta-analysis (knowledge)
Fok, 2024 [17] (Canada)	Undergraduate nursing students; single-group pre–post pilot (*n* = 23–24)	Online module with virtual gaming simulation; darker skin tones	None	Knowledge, PZ-PIKT (0–72); attitudes, APuP; classification (10 photographs); SET-M; immediately post-module	Uncontrolled	Narrative: no concurrent comparator

*n* = intervention/comparator analysed. Excluded on re-assessment (physical simulation without a digital component; used in a sensitivity analysis only): Erdem Onder and Sari, 2025 [16] (Türkiye; three-arm RCT of in situ or laboratory moulage on a standardized patient vs. a pressure injury simulator). “Meta-analysis” refers to the continuous knowledge outcome only. APuP, Attitude towards Pressure Ulcer Prevention instrument; ICU, intensive care unit; NIS, nursing information system; PUKT, Pieper Pressure Ulcer Knowledge Test; PZ-PIKT/PZ-PUKT, Pieper–Zulkowski Pressure Injury/Ulcer Knowledge Test; RCT, randomized controlled trial; SET-M, Simulation Effectiveness Tool—Modified; TEL, technology-enhanced learning.

**Table 2:** Sensitivity analyses for post-intervention pressure injury knowledge.

Analysis	Studies	Hedges’ *g* (95% CI)	I^2^
Primary analysis (DerSimonian–Laird random effects)	3	1.33 (0.98 to 1.69)	41.5%
HKSJ-adjusted confidence interval	3	1.33 (0.55 to 2.12)	–
Paule–Mandel estimator of τ^2^	3	1.34 (0.98 to 1.69)	–
Common-effect (fixed-effect) model	3	1.30 (1.04 to 1.56)	–
Omitting Morente et al. [13]	2	1.17 (0.87 to 1.47)	0%
Omitting Karimian et al. [15] (immediate post-tests only)	2	1.41 (0.81 to 2.00)	70.0%
Omitting Chao et al. [6] (randomized trials only)	2	1.49 (0.98 to 1.99)	43.7%
Adding excluded physical-simulation trial (Erdem Onder and Sari, in situ arm)	4	1.19 (0.80 to 1.58)	61.7%
Adding Erdem Onder and Sari, both moulage arms combined	4	1.12 (0.60 to 1.63)	79.9%

HKSJ, Hartung–Knapp–Sidik–Jonkman.

**Table 3:** GRADE summary of findings: TEL compared with comparator education for post-intervention pressure injury knowledge.

GRADE Item	Assessment
Outcome	Post-intervention pressure injury knowledge (assessed immediately to 3 months after the intervention)
Studies (participants)	3 (277): 2 RCTs and 1 non-randomized matched comparison
Effect (95% CI)	Hedges’ *g* 1.33 (0.98 to 1.69); HKSJ-adjusted 0.55 to 2.12
Starting certainty	High: two RCTs and one non-randomized study assessed with ROBINS-I, whose limitations are captured under risk of bias (GRADE guidance [30])
Risk of bias	Serious (−1): one RCT at high risk; the non-randomized study at serious risk; the remaining RCT with some concerns (allocation concealment, no prespecified analysis plan)
Inconsistency	Not serious: I^2^ = 41.5%, Q *p* = 0.18; all estimates favour TEL, with overlapping CIs
Indirectness	Not serious: all studies enrolled the target populations, measured the primary outcome, and used comparators within the prespecified set; varied comparators were addressed by interpreting the effect as a package effect and by sensitivity analyses. For the narrower question of the digital component alone, only one study provides direct evidence, and certainty would be very low.
Imprecision	Serious (−1): CI excludes small effects, but N = 277 (131/146) is below the optimal information size of about 175 per group (two-sided α 0.05, power 80%, minimal important difference 0.3 SD); HKSJ-adjusted CI 0.55 to 2.12
Publication bias	Not assessable with three studies; not downgraded
Certainty of evidence	⊕⊕◯◯ Low (randomized trials only: *g* 1.49, 95% CI 0.98 to 1.99; 139 participants; also low)

The full evidence profile is given in Supplementary Table S9. Certainty is rated down one level per serious concern. CI, confidence interval; HKSJ, Hartung–Knapp–Sidik–Jonkman; RCT, randomized controlled trial. A *g* of 1.33 corresponds to a large, standardized difference. ⊕⊕◯◯, low certainty: confidence in the effect estimate is limited, and the true effect may be substantially different from the estimate.

## Data Availability

All data and code needed to reproduce the reported results are provided with this article: study-level input data in Supplementary Table S2 (File S1, and File S2 with live formulas), study-level calculations in Supplementary Table S6, and the complete analysis code in Code S1 (File S1, and File S3 as a script).

## Supplementary Materials

The following supporting information can be downloaded at https://www.mdpi.com/article/10.3390/nursrep16100371/s1, File S1 (Supplementary Material, one document): Table S1: Search strategies, final search dates, limits, and records retrieved; Table S2: Data used in the meta-analysis; Table S3: RoB 2 assessments; Table S4: ROBINS-I assessment; Table S5: NHLBI quality assessment; Table S6: Study-level effect-size calculations; Table S7: Sensitivity analyses; Table S8: Studies that might appear to meet the inclusion criteria but were excluded; Table S9: GRADE evidence profile; Table S10: PRISMA 2020 checklist; Figure S1: RoB 2 judgments; Figure S2: ROBINS-I judgments; Figure S3: Leave-one-out analysis; Figure S4: Search-history records of the final searches; Code S1: complete analysis code. File S2: Supplementary Table S2, extraction matrix with live effect-size calculations; Table S6 (live calculation), Hedges’ g and DerSimonian–Laird random-effects meta-analysis (Excel). File S3: Code S1, analysis script (Python).

## Public Involvement Statement

No public involvement in any aspect of this research.

## Guidelines and Standards Statement

This manuscript was drafted in accordance with the PRISMA 2020 statement for systematic reviews and meta-analyses [19].

## Abbreviations

APuP	Attitude towards Pressure Ulcer Prevention instrument
CENTRAL	Cochrane Central Register of Controlled Trials
CI	confidence interval
GRADE	Grading of Recommendations Assessment, Development and Evaluation
HKSJ	Hartung–Knapp–Sidik–Jonkman
ICU	intensive care unit
NHLBI	National Heart, Lung, and Blood Institute
NIS	nursing information system
PICOS	population, intervention, comparator, outcome, study design
PRISMA	Preferred Reporting Items for Systematic Reviews and Meta-Analyses
PUKT	Pieper Pressure Ulcer Knowledge Test
PZ-PIKT/PZ-PUKT	Pieper–Zulkowski Pressure Injury/Ulcer Knowledge Test
RCT	randomized controlled trial
RoB 2	Cochrane Risk of Bias tool for randomized trials, version 2
ROBINS-I	Risk Of Bias In Non-randomized Studies of Interventions
SET-M	Simulation Effectiveness Tool—Modified
TEL	technology-enhanced learning
