# Supplementary material for: Technology-Enhanced Learning for Pressure Injury Prevention in Nursing: A Systematic Review and Meta-Analysis
Source: Nurs Rep. 2026 Oct 20;16(10):371. doi: 10.3390/nursrep16100371 (PMC13708393; doi:10.3390/nursrep16100371)
Supplement: Supplementary file 1 [file nursrep-16-00371-s001.zip › nursrep-4584797-File S1.pdf]

Supplementary Material

File S1 — Technology-Enhanced Learning for Pressure Injury Prevention in Nursing: A Systematic Review and Meta-Analysis

Contents of File S1

- Table S1. Search strategies, final search dates, limits, and records retrieved
- Figure S4. Search-history records of the final searches (7 October 2026)
- Table S2. Data used in the meta-analysis (summary; full extraction matrix in File S2)
- Table S3. RoB 2 assessments (randomized trials)
- Table S4. ROBINS-I assessment (non-randomized comparison)
- Table S5. NHLBI quality assessment (single-group pre–post study)
- Table S6. Study-level effect-size calculations
- Table S7. Sensitivity analyses
- Table S8. Studies that might appear to meet the inclusion criteria but were excluded
- Table S9. GRADE evidence profile
- Figure S1. RoB 2 judgments
- Figure S2. ROBINS-I judgments
- Figure S3. Leave-one-out analysis
- Code S1. Complete analysis code (also provided as File S3)
- Table S10. PRISMA 2020 checklist

Accompanying files. File S2: Supplementary Table S2, full extraction matrix with live effect-size calculations (Excel). File S3: Code S1, analysis script (Python).

Table S1. Search strategies, final search dates, limits, and records retrieved.

Database (platform)	Search dates	Limits	Records	Search strategy as run
MEDLINE (PubMed)	First search 4 April 2026; **final search 7 October 2026**	Publication date 2000/01/01–2026/10/07	1,424	(“Pressure Ulcer”[Mesh] OR “pressure ulcer*”[tiab] OR “pressure injur*”[tiab] OR “pressure sore*”[tiab] OR “decubitus ulcer*”[tiab] OR bedsore*[tiab] OR “bed sore*”[tiab]) AND (“Education, Nursing”[Mesh] OR “Computer-Assisted Instruction”[Mesh] OR “Simulation Training”[Mesh] OR “Patient Simulation”[Mesh] OR educat*[tiab] OR teach*[tiab] OR train*[tiab] OR learn*[tiab] OR simulat*[tiab] OR moulage[tiab] OR “standardized patient*”[tiab] OR “standardised patient*”[tiab] OR multimedia[tiab] OR “serious game*”[tiab]) AND (“Nurses”[Mesh] OR “Students, Nursing”[Mesh] OR “Nursing Assistants”[Mesh] OR nurs*[tiab]) AND (“2000/01/01”[Date - Publication] : “2026/10/07”[Date - Publication])
Scopus (Elsevier)	First search January 2026 (10–24 January); **final search 7 October 2026**	PUBYEAR > 1999 AND PUBYEAR < 2027	2,120	TITLE-ABS-KEY(“pressure ulcer*” OR “pressure injur*” OR “pressure sore*” OR “decubitus ulcer*” OR bedsore* OR “bed sore*”) AND TITLE-ABS-KEY(educat* OR teach* OR train* OR learn* OR simulat* OR moulage OR multimedia OR “standardized patient*” OR “standardised patient*” OR “serious game*”) AND TITLE-ABS-KEY(nurs*) AND PUBYEAR > 1999 AND PUBYEAR < 2027
CINAHL (EBSCOhost)	First search January 2026 (10–24 January); **final search 7 October 2026**	Publication date 2000–2026	351	S1: (MH “Pressure Ulcer+”) OR TI (“pressure ulcer*” OR “pressure injur*” OR “pressure sore*” OR “decubitus ulcer*” OR bedsore* OR “bed sore*”) OR AB (“pressure ulcer*” OR “pressure injur*” OR “pressure sore*” OR “decubitus ulcer*” OR bedsore* OR “bed sore*”); S2: TI (educat* OR teach* OR train* OR learn* OR simulat* OR moulage OR multimedia OR “standardized patient*” OR “standardised patient*” OR “serious game*”) OR AB (same terms); S3: TI nurs* OR AB nurs*; S4: S1 AND S2 AND S3
Cochrane CENTRAL (Cochrane Library, Issue 10 of 12, October 2026)	**Final search 7 October 2026**	Year first published 2000–2026 (311 → 300 trial records); 6 Cochrane Reviews not exported	300	#1 [mh “Pressure Ulcer”]; #2 ((pressure NEXT ulcer*) OR (pressure NEXT injur*) OR (pressure NEXT sore*) OR (decubitus NEXT ulcer*) OR bedsore* OR (bed NEXT sore*)):ti,ab,kw; #3 (educat* OR teach* OR train* OR learn* OR simulat* OR moulage OR multimedia OR (standardized NEXT patient*) OR (standardised NEXT patient*) OR (serious NEXT game*)):ti,ab,kw; #4 (nurs*):ti,ab,kw; #5 (#1 OR #2) AND #3 AND #4
**Total**			**4,195**	Duplicates removed: 2,146; records screened: 2,049

Note. Record counts are those of the final searches run on 7 October 2026, which identified no additional eligible studies compared with the first searches. Strategies are reproduced verbatim from each database’s search history; screenshots of the final searches are shown in Figure S4. These searches replace the search strategies reported in the first submission, which had been reconstructed because the original search histories were not retained. Line counts in CENTRAL: #1 1,230; #2 3,152; #3 359,566; #4 71,964; #5 317 (311 trials and 6 Cochrane Reviews).

History and Search Details

Download Delete

Search	Actions	Details	Query	Results	Time
#3	...		Search: **("Pressure Ulcer"[Mesh] OR "pressure ulcer*"[tiab] OR "pressure injur*"[tiab] OR "pressure sore*"[tiab] OR "decubitus ulcer*"[tiab] OR bedsore*[tiab] OR "bed sore*"[tiab]) AND ("Education, Nursing"[Mesh] OR "Computer-Assisted Instruction" [Mesh] OR "Simulation Training"[Mesh] OR "Patient Simulation" [Mesh] OR educat*[tiab] OR teach*[tiab] OR train*[tiab] OR learn* [tiab] OR simulat*[tiab] OR moulage[tiab] OR "standardized patient*"[tiab] OR "standardised patient*"[tiab] OR multimedia[tiab] OR "serious game*"[tiab]) AND ("Nurses" [Mesh] OR "Students, Nursing"[Mesh] OR "Nursing Assistants" [Mesh] OR nurs*[tiab]) AND ("2000/01/01"[Date - Publication] : "2026/10/07"[Date - Publication])**<br>("Pressure Ulcer"[MeSH Terms] OR "pressure ulcer*"[Title/Abstract] OR "pressure injur*"[Title/Abstract] OR "pressure sore*" [Title/Abstract] OR "decubitus ulcer*"[Title/Abstract] OR "bedsore*" [Title/Abstract] OR "bed sore*"[Title/Abstract]) AND ("education, nursing"[MeSH Terms] OR "Computer-Assisted Instruction"[MeSH Terms] OR "Simulation Training"[MeSH Terms] OR "Patient Simulation"[MeSH Terms] OR "educat*"[Title/Abstract] OR "teach*" [Title/Abstract] OR "train*"[Title/Abstract] OR "learn*"[Title/Abstract] OR "simulat*"[Title/Abstract] OR "moulage"[Title/Abstract] OR "standardized patient*"[Title/Abstract] OR "standardised patient*" [Title/Abstract] OR "multimedia"[Title/Abstract] OR "serious game*" [Title/Abstract]) AND ("Nurses"[MeSH Terms] OR "students, nursing" [MeSH Terms] OR "Nursing Assistants"[MeSH Terms] OR "nurs*" [Title/Abstract]) AND 2000/01/01:2026/10/07[Date - Publication]	1,424	03:34:40

Figure S4a. *Search-history records of the final searches (7 October 2026). MEDLINE via PubMed, search history: search #3, 1,424 records (publication dates 2000/01/01–2026/10/07).*

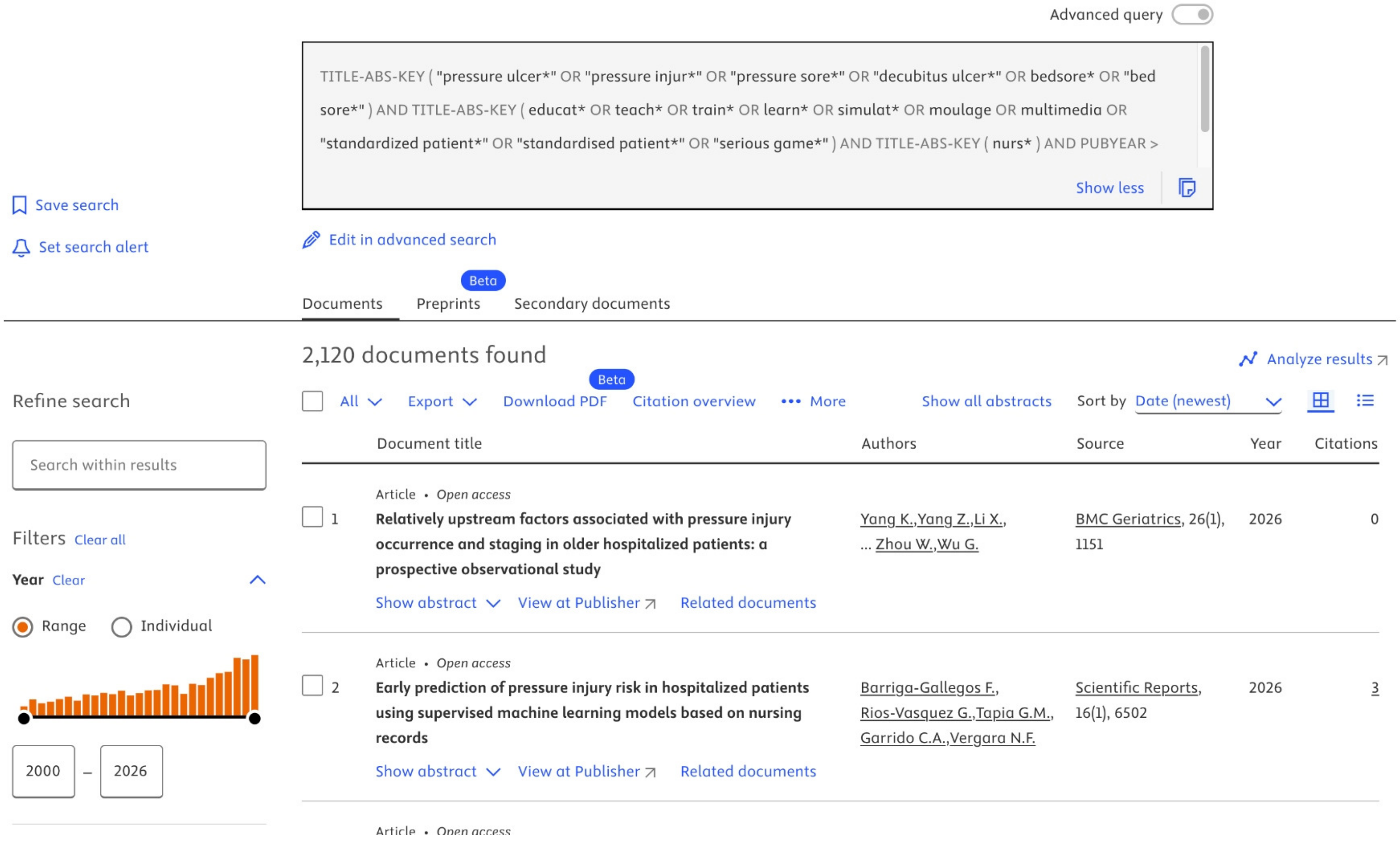

Figure S4b. *Scopus, advanced query with publication years 2000–2026: 2,120 documents.*

Save search

Search name

(MH "Pressure Ulcer+")OR TI ("pressure ulcer*" OR "pressure injur*" OR "pressure sore*" OR "decub

255/255

Description (optional)

0/300

Query

(MH "Pressure Ulcer+")
OR TI ("pressure ulcer*" OR "pressure injur*" OR "pressure sore*" OR "decubitus ulcer*" OR bedsore* OR "bed sore*")
OR AB ("pressure ulcer*" OR "pressure injur*" OR "pressure sore*" OR "decubitus ulcer*" OR bedsore* OR "bed sore*") AND (TI (educat* OR teach* OR train* OR learn* OR simulat* OR moulage OR multimedia OR "standardized patient*" OR "standardised patient*" OR "serious game*")
OR AB (educat* OR teach* OR train* OR learn* OR simulat* OR moulage OR multimedia OR "standardized patient*" OR "standardised patient*" OR "serious game*")) AND (TI nurs* OR AB nurs*)

Copy to clipboard

Results

351

Figure S4c. *CINAHL (EBSCOhost), saved search: query lines and result (351 records). The 2000–2026 publication-date limiter was applied in the search interface and is not displayed in this dialog.*

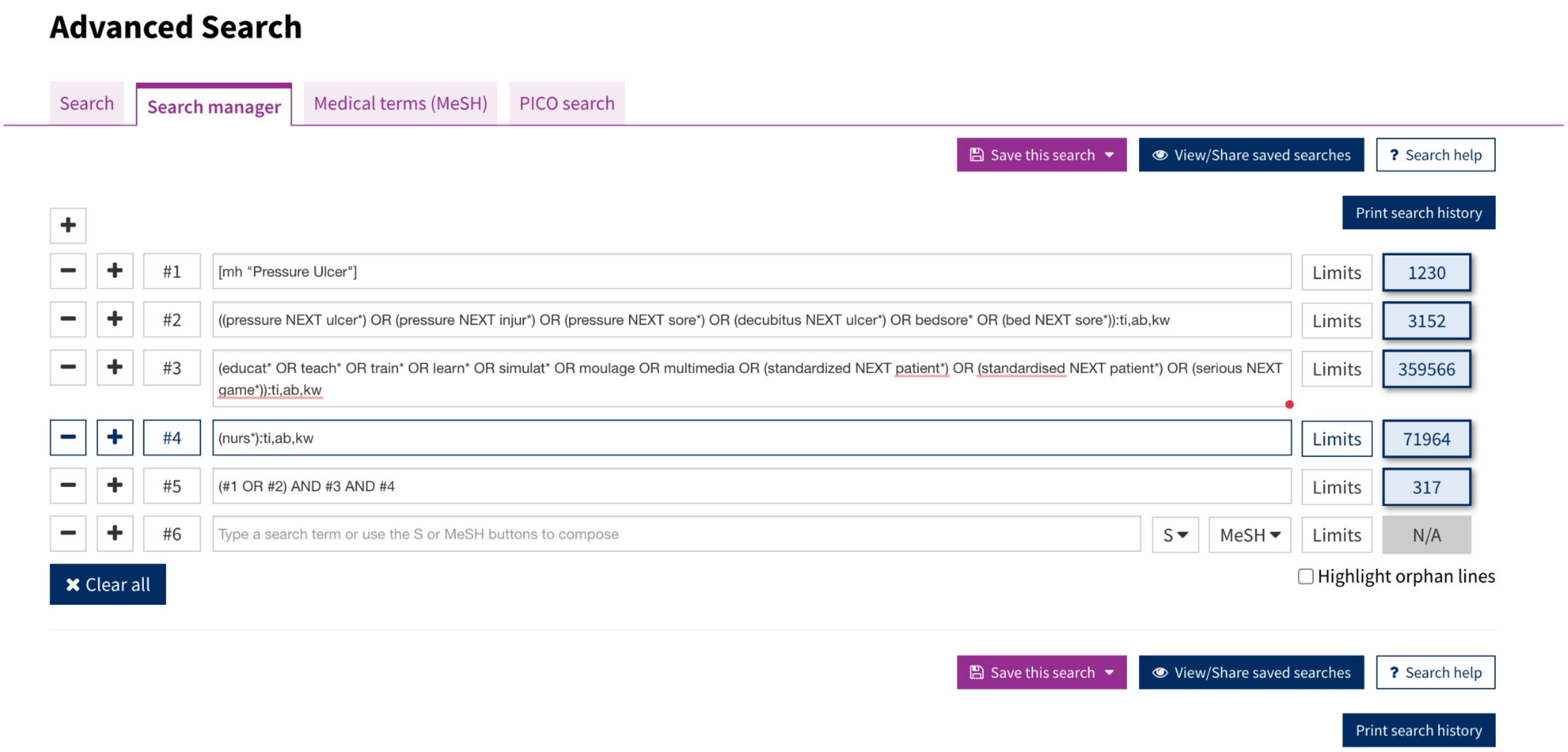

Figure S4d. *Cochrane Library, CENTRAL (Issue 10 of 12, October 2026): search #5 = (#1 OR #2) AND #3 AND #4 returned 311 trial records and 6 Cochrane Reviews; the year-first-published limit (2000–2026) reduced the trial records to 300, which were exported.*

Table S2. Data used in the meta-analysis. The full extraction matrix (10 outcome records from 6 studies, including the excluded study used in a sensitivity analysis, with page and table sources) is provided in File S2 (Excel), which also recalculates every effect size and the pooled estimate with live formulas.

Study	Outcome; instrument (range)	Time point	TEL n	TEL mean (SD)	Comparator n	Comparator mean (SD)	Source in report
Morente et al., 2014	Applied evaluation knowledge; image-based questionnaire (0–22)	Immediately post-session	30	15.83 (2.39)	42	11.60 (2.39)	Tables 1–2, pp. 2047–2048 (abstract reports control SD 2.52; table value used)
Karimian et al., 2020	Knowledge; PUKT (0–47)	3 months	32	29.75 (12.33)	35	17.54 (6.62)	Table 2, p. 93
Chao et al., 2025	Knowledge; PZ-PUKT (0–72)	Immediately post-workshop	69	53.44 (6.66)	69	45.10 (7.85)	Table 2, p. 7 (propensity-score-matched pairs)
Erdem Onder & Sari, 2025 (excluded; sensitivity only)	Knowledge; PIKT (0–100)	Post-simulation	28	82.85 (10.66)	28	76.25 (8.98)	Table 2, p. 7 (in-situ moulage arm vs simulator control)
Erdem Onder & Sari, 2025 (excluded; sensitivity only)	Knowledge; PIKT (0–100)	Post-simulation	28	78.03 (11.73)	28	76.25 (8.98)	Table 2, p. 7 (laboratory moulage arm)

Table S3. RoB 2 assessments for randomized trials, by outcome and time point.

Study; outcome	Domain	Judgment	Supporting rationale
**Morente 2014**; Applied evaluation knowledge, immediate post-test	D1 Randomization process	Some concerns	Individual randomization using a computer-generated sequence; allocation concealment not reported. Baseline knowledge scores comparable (8.27 vs 8.23).
	D2 Deviations from intended interventions	Low	Participants and educators could not be blinded, but both arms received 4 hours of instruction using the same slide content; no deviations reported. Effect of assignment analysed.
	D3 Missing outcome data	Low	72 of 73 randomized participants (98.6%) assessed post-test.
	D4 Measurement of the outcome	Low	Objective, researcher-developed image-based questionnaire with fixed scoring; scoring not dependent on assessor judgement, although assessor blinding not reported.
	D5 Selection of the reported result	Some concerns	No registered protocol or prespecified analysis plan identified; a single total score was reported. The abstract and results table report different control-group SDs (2.52 vs 2.39); the table value was used.
	Overall	**Some concerns**	Some concerns in at least one domain without any domain at high risk.
**Bredesen 2016**; Classification performance, immediate post-test	D1 Randomization process	Some concerns	Block randomization reported; method of allocation concealment not described.
	D2 Deviations from intended interventions	Low	Both arms received identical content; no deviations reported.
	D3 Missing outcome data	Some concerns	44 of 50 allocated participants (88%) completed the immediate assessment; reasons for missingness not fully reported. (The 3-month follow-up, n = 10/8, was judged High risk for this domain and is interpreted narratively only.)
	D4 Measurement of the outcome	Low	Photograph-based classification against a reference standard; objective scoring.
	D5 Selection of the reported result	Some concerns	No prespecified analysis plan identified; outcomes reported as proportions of photograph responses rather than participant-level scores.
	Overall	**Some concerns**	Some concerns in at least one domain without any domain at high risk.
**Karimian 2020**; Knowledge (PUKT), 3 months post-intervention	D1 Randomization process	Some concerns	Allocation to experimental and control groups reported as randomized, but sequence generation and concealment are not clearly described. Baseline knowledge comparable (15.68 vs 16.40).
	D2 Deviations from intended interventions	Some concerns	Control condition not clearly specified; contamination between ICU nurses on shared units cannot be excluded.
	D3 Missing outcome data	Some concerns	Group sizes 32/35 analysed; outcome-specific attrition and handling of missing data not reported.
	D4 Measurement of the outcome	Some concerns	Self-administered knowledge test completed by unblinded participants who knew their allocation; assessment at 3 months.
	D5 Selection of the reported result	Some concerns	No registered protocol identified. The intervention-group SD at follow-up (12.33 on a 0–47 scale) is large relative to baseline (3.42) and was not explained.
	Overall	**High**	Multiple domains with some concerns substantially lower confidence in the result (RoB 2 guidance).
**Erdem Onder 2025**†; Knowledge	D1 Randomization process	Low	Stratified block randomization by GPA using a computer-generated sequence; baseline comparability

Study; outcome	Domain	Judgment	Supporting rationale
(PIKT), post-simulation			reported.
	D2 Deviations from intended interventions	Some concerns	Blinding of students and facilitators not possible; all arms received theoretical education and active simulation, but fidelity and deviations across simulation settings were not fully reported.
	D3 Missing outcome data	Low	84 of 90 randomized students (93%) completed; 28 per arm analysed.
	D4 Measurement of the outcome	Low	15-item multiple-choice test with objective scoring.
	D5 Selection of the reported result	Some concerns	Trial registration retrospective; the results table reports an omnibus three-group Kruskal–Wallis test, with pairwise comparisons summarised without exact p values.
	Overall	**Some concerns**	Some concerns in at least one domain without any domain at high risk.

Note. †Erdem Onder & Sari (2025) was excluded from the review (physical simulation without a digital component) and is assessed here only because it is used in a sensitivity analysis. D3 for the Bredesen et al. three-month follow-up (10 and 8 participants) was judged high risk; that time point contributes only to the narrative synthesis.

Table S4. ROBINS-I assessment for the non-randomized comparison.

Study; outcome	Domain	Judgment	Supporting rationale
**Chao 2025**; Knowledge (PZ-PUKT), immediate post-test	D1 Confounding	Serious	Allocation followed participant availability on days off (partially randomized participant-preference design). Propensity-score matching balanced measured covariates only; residual confounding by motivation and unit factors is likely.
	D2 Selection of participants	Serious	Of 218 enrolled nurses, 79 completed all measurements; the analysis used 69 matched pairs, so selection into the analysis was related to completion and matching.
	D3 Classification of interventions	Low	Intervention groups (digital manual plus multimedia and workshop vs digital manual alone) clearly defined at the start.
	D4 Deviations from intended interventions	Moderate	Both groups had access to the digital manual; co-exposure and adherence to the self-study component were not measured.
	D5 Missing data	Serious	Substantial non-completion; analysis restricted to matched completers.
	D6 Measurement of outcomes	Moderate	Self-administered, validated knowledge test; participants aware of their group.
	D7 Selection of the reported result	Moderate	Matched-pair analysis reported; no registered analysis plan identified. The matched design was analysed here with an independent-groups variance because within-pair correlation was not reported.
	Overall	**Serious**	Serious risk in at least one domain (confounding, selection, missing data); no domain at critical risk.

Table S5. NHLBI Quality Assessment Tool for Before–After (Pre–Post) Studies With No Control Group: Fok (2024).

No.	Criterion (abbreviated)	Assessment	Supporting rationale
1	Clear study objective	Yes	The objective was to evaluate changes in nursing students' knowledge, attitudes, and classification skills after an online educational module.
2	Eligibility criteria prespecified and described	Yes	Target population and eligibility criteria for undergraduate nursing students in British Columbia are specified.
3	Participants representative of the population of interest	No	Participation was voluntary; students from some regions were not represented, and recruitment coverage across nursing schools was uncertain.
4	All eligible participants enrolled	Cannot determine	The number of eligible students who received the invitation was not established.
5	Sample size sufficient	No	The calculated requirement was 34 participants; 23 contributed complete knowledge data.
6	Intervention clearly described and delivered consistently	Yes	Participants accessed a defined online module with a virtual gaming simulation ("Clara's Story").
7	Outcome measures prespecified, valid, reliable, consistently applied	Yes	Knowledge was assessed before and after with the Pieper–Zulkowski Pressure Injury Knowledge Test, with psychometric information reported.
8	Outcome assessors blinded	Not reported	Blinded outcome assessment was not reported; online scoring limits assessor discretion but does not establish blinding.
9	Loss to follow-up $\leq$ 20%; losses accounted for	Cannot determine	Extensive survey non-completion was documented, but the number completing the baseline knowledge test and missing the post-test was not clearly isolated.
10	Appropriate statistical analysis of pre–post change	Yes	Paired Wilcoxon signed-rank tests (knowledge, attitudes) and a paired t-test (classification) were used.
11	Multiple outcome measurements before and after	No	One pre-test and one immediate post-test only; no repeated baseline or longer-term assessment.
12	Group-level intervention accounted for	Not applicable	Individual-level intervention and analysis.
—	**Overall quality**	**Fair**	Voluntary recruitment, a sample below the calculated requirement, incomplete data, and single pre- and post-measurements weaken confidence; the uncontrolled design cannot separate intervention effects from testing or maturation effects.

Table S6. Study-level effect-size calculations (Hedges' g; DerSimonian–Laird random-effects weights).

Study	n_1	n_2	Pooled SD	Cohen's *d*	*J*	Hedges' *g*	Variance	SE	95% CI	Weight
Morente et al., 2014	30	42	2.390	1.770	0.9892	1.751	0.0784	0.280	1.20 to 2.30	27.3%
Karimian et al., 2020	32	35	9.769	1.250	0.9884	1.235	0.0712	0.267	0.71 to 1.76	29.1%
Chao et al., 2025	69	69	7.279	1.146	0.9945	1.139	0.0337	0.184	0.78 to 1.50	43.6%
**Pooled (random effects)**	131	146				**1.334**		0.181	**0.98 to 1.69**	100%

Formulas. Pooled SD = $\sqrt{\{[(n_1-1)SD_1^2 + (n_2-1)SD_2^2]/(n_1+n_2-2)\}}$; $d = (M_1 - M_2)$/pooled SD; $J = 1 - 3/(4[n_1+n_2] - 9)$; $g = J \cdot d$; $\text{Var}(g) = (n_1+n_2)/(n_1 n_2) + g^2/(2[n_1+n_2])$. Heterogeneity: Q = 3.42, df = 2, p = 0.181; I^2 = 41.5%; τ^2 (DerSimonian–Laird) = 0.0410; z = 7.39, p < 0.001; 95% prediction interval -2.11 to 4.78 (one degree of freedom; uninformative). Results were identical to three decimal places when recomputed with statsmodels 0.15 (*combine_effects*, method "dl"). Paule–Mandel τ^2 = 0.044 gave *g* = 1.34 (95% CI 0.98 to 1.69).

Subgroups (descriptive). Nursing students (one study, Morente et al.): *g* = 1.75 (95% CI 1.20 to 2.30). Registered nurses (two studies): *g* = 1.17 (95% CI 0.87 to 1.47), I^2 = 0%, τ^2 = 0. Between-subgroup Q = 3.328, df = 1, p = 0.07.

Table S7. Sensitivity analyses.

Analysis	k	Hedges' *g* (95% CI)	I^2	τ^2
Primary: DerSimonian–Laird, Wald CI	3	1.33 (0.98 to 1.69)	41.5%	0.041
HKSJ-adjusted CI (t, df = 2)	3	1.33 (0.55 to 2.12); p = 0.018	–	–
Paule–Mandel τ^2	3	1.34 (0.98 to 1.69)	–	0.044
Common-effect model	3	1.30 (1.04 to 1.56)	–	–
Omitting Morente et al., 2014	2	1.17 (0.87 to 1.47)	0.0%	0.000
Omitting Karimian et al., 2020	2	1.41 (0.81 to 2.00)	70.0%	0.131
Omitting Chao et al., 2025	2	1.49 (0.98 to 1.99)	43.7%	0.058
Adding excluded trial Erdem Onder & Sari (in-situ moulage arm vs simulator control; study *g* = 0.66, 0.12 to 1.20)	4	1.19 (0.80 to 1.58)	61.7%	0.098
Adding Erdem Onder & Sari, in-situ and laboratory moulage arms combined (n = 56; mean 80.44, SD 11.37; study *g* = 0.39, -0.07 to 0.85)	4	1.12 (0.60 to 1.63)	79.9%	0.222

Note. Omitting Chao et al. restricts the analysis to randomized trials; omitting Karimian et al. restricts it to immediate post-intervention assessments. Arms were combined using the formula in the Cochrane Handbook (Table 6.5.a).

Table S8. Studies that might appear to meet the inclusion criteria but were excluded.

Study	Design and population	Intervention vs comparator	Stage excluded	Reason for exclusion
Erdem Onder H, Sari D. BMC Nurs. 2025;24:1373	Three-arm RCT; senior undergraduate nursing students (n = 84)	In-situ or laboratory moulage on a standardized patient vs pressure injury simulator on a standardized patient	Full text (re-assessed during peer review)	Physical simulation without a digital component; not TEL. Used in a sensitivity analysis.
Baran Z, Ayik C, Özden D. Nurse Educ Today. 2025;151:106735	RCT; nursing students (n = 79)	Standardized patient vs medium-fidelity manikin simulation	Title/abstract	Physical simulation without a digital component; not TEL.
Altun S, Tastan S. J Tissue Viability. 2022;31(4):643–648	RCT; nursing students (n = 81)	Standardized patient vs low-fidelity manikin simulator	Title/abstract	Physical simulation without a digital component; not TEL.
Çakar V, Karadağ A. Simul Gaming. 2024;55(4):576–599	Quasi-experimental; patient relatives	Serious game on pressure injury prevention	Full text	Population: not nurses or nursing students.

Table S9. GRADE evidence profile for post-intervention pressure injury knowledge (TEL vs comparator education).

GRADE domain	All controlled studies (primary analysis)	Randomized trials only (check)
Studies (participants)	3 (277; 131 TEL, 146 comparator): Morente 2014 and Karimian 2020 (RCTs); Chao 2025 (non-randomized, propensity-score matched)	2 (139): Morente 2014; Karimian 2020
Effect, Hedges' *g* (95% CI)	1.33 (0.98 to 1.69); HKSJ 0.55 to 2.12	1.49 (0.98 to 1.99)
Starting certainty	High. The non-randomized study was assessed with ROBINS-I; following GRADE guidance 18, evidence assessed with ROBINS-I may start at high certainty, with confounding and selection bias captured in the risk-of-bias domain.	High (randomized trials).
Risk of bias	Serious (−1). Karimian 2020 hi□h risk (RoB 2: some concerns in all five domains); Chao 2025 serious risk (ROBINS-I: confounding, selection, missing data); Morente 2014 some concerns (allocation concealment, no prespecified analysis plan). Studies at high or serious risk carry 72.7% of the weight.	Serious (−1). Karimian 2020 high risk; Morente 2014 some concerns.
Inconsistency	Not serious. $I^2 = 41.5\%$, $\tau^2 = 0.041$, Q $p = 0.18$; all three estimates favour TEL and their CIs overlap.	Not serious. Both estimates large and favouring TEL; $I^2 = 43.7\%$.
Indirectness	Not serious for the review question (TEL vs any prespecified comparator): target populations (nursing students, registered nurses), the primary outcome (knowledge), and comparators within the prespecified set. Comparators differed (lecture with identical content; no specified education; digital manual alone); this is addressed by interpreting the estimate as the effect of TEL-based educational packages and by sensitivity analyses. *For the narrower question of whether the digital component itself improves knowledge, only Morente 2014 provides a direct comparison; evidence for that question would be rated down for indirectness (very low certainty).*	As for the primary analysis.
Imprecision	Serious (−1). The 95% CI excludes small effects, but GRADE recommends considering the optimal information size (OIS) when a large effect comes from a small sample (GRADE guidance 6). OIS assumptions: two-sided $\alpha = 0.05$, power = 80%, minimal important difference = 0.3 SD, giving $n = 2(z_{0.975} + z_{0.80})^2/0.3^2 \approx 175$ per group (350 in total). Observed: 131 and 146 per group. The HKSJ-adjusted CI (0.55 to 2.12) is wide.	Serious (−1). 139 participants (62 and 77 per group), well below the OIS.
Publication bias	Not assessable with three studies (funnel-plot tests require at least ten); not rated down.	Not assessable.
Large effect (upgrading)	Not applied: the evidence had serious risk-of-bias concerns, and the large effect may partly reflect bias, short-term content-aligned tests, and a no-education comparator (manuscript Section 4.2).	Not applied.
**Certainty**	⊕⊕◯◯ **Low**	⊕⊕◯◯ **Low**

Note. Minimal important difference of 0.3 SD chosen as a small-to-moderate educational effect; with 0.2 SD the OIS is about 393 per group, and with 0.5 SD about 63 per group. The OIS criterion is not met for any minimal important difference below about 0.35 SD. CI, confidence interval; HKSJ, Hartung–Knapp–Sidik–Jonkman; RCT, randomized controlled trial; SD, standard deviation; TEL, technology-enhanced learning.

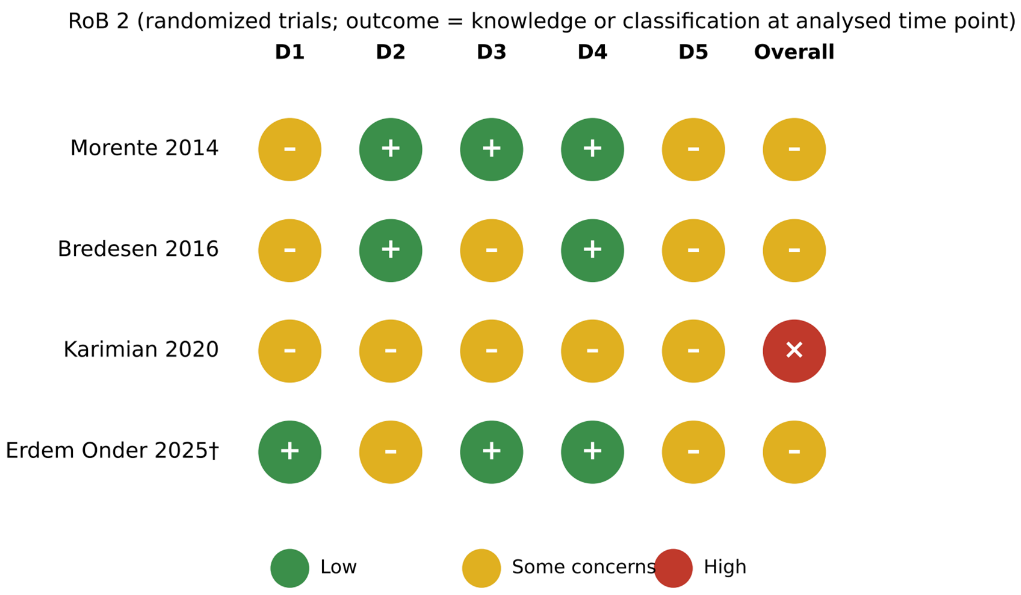

Figure S1. *RoB 2 judgments for randomized trials. D1, randomization process; D2, deviations from intended interventions; D3, missing outcome data; D4, measurement of the outcome; D5, selection of the reported result. †Excluded from the review; assessed only because it is used in a sensitivity analysis.*

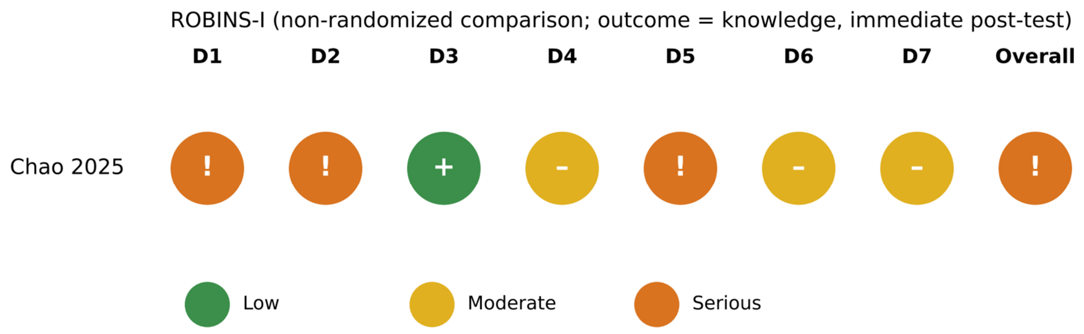

Figure S2. *ROBINS-I judgments for Chao et al. (2025). D1, confounding; D2, selection of participants; D3, classification of interventions; D4, deviations from intended interventions; D5, missing data; D6, measurement of outcomes; D7, selection of the reported result.*

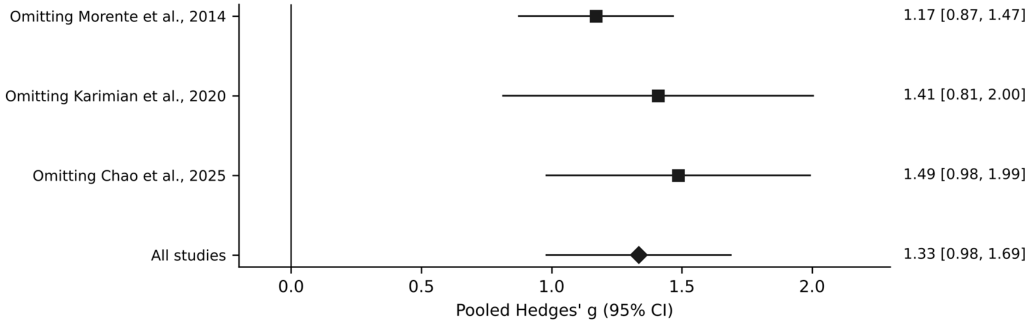

Figure S3. *Leave-one-out sensitivity analysis (random effects, Hedges' g).*

Code S1. Complete analysis code (Python 3.13; NumPy 2.5, SciPy 1.18, statsmodels 0.15). Running the script reproduces every number in the main text, Tables 2–3, and Tables S6, S7, and S9 from the data in Table S2. The same script is provided as File S3.

```python
"""Reproducible meta-analysis for: Technology-Enhanced Learning for Pressure Injury
Prevention in Nursing: A Systematic Review and Meta-Analysis. Data = Supplementary Table S2.
Effect size: Hedges' g (pooled-SD SMD with small-sample correction J).
Pooling: random effects, DerSimonian-Laird tau^2; HKSJ, Paule-Mandel and common-effect
models as sensitivity analyses.
Primary analysis: the three controlled studies meeting the digital-TEL criterion.
Eligibility sensitivity: adding Erdem Onder & Sari (2025), excluded because it evaluated physical
simulation without a digital component."""

import json, math
import numpy as np
from scipy.stats import chi2, norm, t
from statsmodels.stats.meta_analysis import combine_effects

STUDIES = [  # label, group, n1, m1, sd1, n2, m2, sd2
    ("Morente et al., 2014", "Nursing students", 30, 15.83, 2.39, 42, 11.60, 2.39),
    ("Karimian et al., 2020", "Registered nurses", 32, 29.75, 12.33, 35, 17.54, 6.62),
    ("Chao et al., 2025", "Registered nurses", 69, 53.44, 6.66, 69, 45.10, 7.85),
]
ERDEM_INSITU = (
    "Erdem Onder & Sari, 2025",
    "Nursing students",
    28,
    82.85,
    10.66,
    28,
    76.25,
    8.98,
)
ERDEM_LAB = (28, 78.03, 11.73)  # second moulage arm, same control


def hedges(n1, m1, s1, n2, m2, s2):
```

```python
    sp = math.sqrt(((n1 - 1) * s1**2 + (n2 - 1) * s2**2) / (n1 + n2 - 2))
    d = (m1 - m2) / sp
    J = 1 - 3 / (4 * (n1 + n2) - 9)
    g = J * d
    v = (n1 + n2) / (n1 * n2) + g**2 / (2 * (n1 + n2))
    return dict(sp=sp, d=d, J=J, g=g, v=v, se=math.sqrt(v))


def pool(ys, vs):
    ys, vs = np.asarray(ys, float), np.asarray(vs, float)
    k = len(ys)
    w = 1 / vs
    fe = (w * ys).sum() / w.sum()
    Q = float((w * (ys - fe) ** 2).sum())
    df = k - 1
    C = w.sum() - (w**2).sum() / w.sum()
    t2 = max(0.0, (Q - df) / C) if k > 1 else 0.0
    wr = 1 / (vs + t2)
    mu = float((wr * ys).sum() / wr.sum())
    se = math.sqrt(1 / wr.sum())
    z = mu / se
    out = dict(
        k=k,
        mu=mu,
        se=se,
        lo=mu - 1.96 * se,
        hi=mu + 1.96 * se,
        z=z,
        p=float(2 * norm.sf(abs(z))),
        Q=Q,
        df=df,
        pQ=float(chi2.sf(Q, df)) if df > 0 else None,
        tau2=t2,
        I2=max(0.0, (Q - df) / Q) * 100 if (Q > 0 and df > 0) else 0.0,
        weights=[float(x) for x in wr / wr.sum() * 100],
        fe=float(fe),
        fe_se=math.sqrt(1 / w.sum()),
```

```python
    )
    if k > 2:  # prediction interval (Riley 2011), t with k-2 df
        tc = t.ppf(0.975, k - 2)
        h = tc * math.sqrt(t2 + se**2)
        out.update(pi_lo=mu - h, pi_hi=mu + h)
    if k > 1:  # HKSJ
        q = float((wr * (ys - mu) ** 2).sum()) / df
        se_h = math.sqrt(q / wr.sum())
        tc = t.ppf(0.975, df)
        out.update(
            hk_lo=mu - tc * se_h,
            hk_hi=mu + tc * se_h,
            hk_p=float(2 * t.sf(abs(mu / se_h), df)),
        )
    return out


def keep(o, keys=("mu", "lo", "hi", "I2", "tau2", "pQ", "k")):
    return {k: o.get(k) for k in keys}


res = {"studies": []}
for lab, grp, *x in STUDIES:
    e = hedges(*x)
    e.update(label=lab, group=grp, n1=x[0], n2=x[3])
    res["studies"].append(e)
ys = [s["g"] for s in res["studies"]]
vs = [s["v"] for s in res["studies"]]
res["overall"] = pool(ys, vs)
for i, s in enumerate(res["studies"]):
    s["weight"] = res["overall"]["weights"][i]
res["N"] = dict(
    int=sum(s["n1"] for s in res["studies"]), ctl=sum(s["n2"] for s in res["studies"])
)

cr = combine_effects(np.array(ys), np.array(vs), method_re="dl")
sf = cr.summary_frame()
```

```python
res["statsmodels_check"] = dict(
    re=float(sf.loc["random effect", "eff"]),
    lo=float(sf.loc["random effect", "ci_low"]),
    hi=float(sf.loc["random effect", "ci_upp"]),
    tau2=float(cr.tau2),
    i2=float(cr.i2),
)
cr2 = combine_effects(np.array(ys), np.array(vs), method_re="iterated")
sf2 = cr2.summary_frame()
res["paule_mandel"] = dict(
    re=float(sf2.loc["random effect", "eff"]),
    lo=float(sf2.loc["random effect", "ci_low"]),
    hi=float(sf2.loc["random effect", "ci_upp"]),
    tau2=float(cr2.tau2),
)

# subgroups (exploratory; nursing students = 1 study)
res["subgroups"] = {}
for grp in ["Nursing students", "Registered nurses"]:
    idx = [i for i, s in enumerate(res["studies"]) if s["group"] == grp]
    sg = pool([ys[i] for i in idx], [vs[i] for i in idx])
    sg["n_int"] = sum(res["studies"][i]["n1"] for i in idx)
    sg["n_ctl"] = sum(res["studies"][i]["n2"] for i in idx)
    sg["N"] = sg["n_int"] + sg["n_ctl"]
    res["subgroups"][grp] = sg
a, b = res["subgroups"]["Nursing students"], res["subgroups"]["Registered nurses"]
mu_w = (a["mu"] / a["se"] ** 2 + b["mu"] / b["se"] ** 2) / (
    1 / a["se"] ** 2 + 1 / b["se"] ** 2
)
Qb = (a["mu"] - mu_w) ** 2 / a["se"] ** 2 + (b["mu"] - mu_w) ** 2 / b["se"] ** 2
res["subgroup_test"] = dict(Qb=Qb, df=1, p=float(chi2.sf(Qb, 1)))

# sensitivity
res["loo"] = []
for i, s in enumerate(res["studies"]):
    o = pool(
        [y for j, y in enumerate(ys) if j != i], [v for j, v in enumerate(vs) if j != i]
```

```
    )
    res["loo"].append(dict(omitted=s["label"], **keep(o)))
e_er = hedges(*ERDEM_INSITU[2:])
res["erdem_insitu"] = dict(
    g=e_er["g"],
    lo=e_er["g"] - 1.96 * e_er["se"],
    hi=e_er["g"] + 1.96 * e_er["se"],
    v=e_er["v"],
)
res["add_erdem_insitu"] = keep(pool(ys + [e_er["g"]], vs + [e_er["v"]]))
n_a, m_a, s_a = ERDEM_INSITU[2:5]
n_b, m_b, s_b = ERDEM_LAB
n_c = n_a + n_b
m_c = (n_a * m_a + n_b * m_b) / n_c
s_c = math.sqrt(
    ((n_a - 1) * s_a**2 + (n_b - 1) * s_b**2 + n_a * n_b / n_c * (m_a - m_b) ** 2)
    / (n_c - 1)
)
e_c = hedges(n_c, m_c, s_c, 28, 76.25, 8.98)
res["erdem_combined"] = dict(
    n=n_c,
    mean=m_c,
    sd=s_c,
    g=e_c["g"],
    lo=e_c["g"] - 1.96 * e_c["se"],
    hi=e_c["g"] + 1.96 * e_c["se"],
)
res["add_erdem_combined"] = keep(pool(ys + [e_c["g"]], vs + [e_c["v"]]))
res["fixed"] = dict(
    mu=res["overall"]["fe"],
    lo=res["overall"]["fe"] - 1.96 * res["overall"]["fe_se"],
    hi=res["overall"]["fe"] + 1.96 * res["overall"]["fe_se"],
)

json.dump(res, open("results.json", "w"), indent=1, default=float)
```

```python
def ci(d, lo="lo", hi="hi"):
    return f"{d['mu']:.2f} ({d[lo]:.2f} to {d[hi]:.2f})"


if __name__ == "__main__":
    print("Study-level effects (Table S6)")
    for s in res["studies"]:
        lo, hi = s["g"] - 1.96 * s["se"], s["g"] + 1.96 * s["se"]
        print(
            f"  {s['label']:24s} g = {s['g']:.3f} ({lo:.2f} to {hi:.2f})"
            f"  var = {s['v']:.4f}  weight = {s['weight']:.1f}%"
        )
    o = res["overall"]
    print(f"Pooled (DL): g = {ci(o)}; z = {o['z']:.2f}; p = {o['p']:.1e}")
    print(
        f"  Q = {o['Q']:.2f}, df = {o['df']}, p = {o['pQ']:.3f}; "
        f"I2 = {o['I2']:.1f}%; tau2 = {o['tau2']:.4f}; N = {res['N']}"
    )
    print(f"  95% PI {o['pi_lo']:.2f} to {o['pi_hi']:.2f}")
    print(f"  HKSJ CI {o['hk_lo']:.2f} to {o['hk_hi']:.2f} (p = {o['hk_p']:.4f})")
    print(f"  Common effect: g = {ci(res['fixed'])}")
    sm, pm = res["statsmodels_check"], res["paule_mandel"]
    print(f"  statsmodels check: g = {sm['re']:.3f} ({sm['lo']:.3f} to {sm['hi']:.3f})")
    print(
        f"  Paule-Mandel: g = {pm['re']:.2f} ({pm['lo']:.2f} to {pm['hi']:.2f}),"
        f" tau2 = {pm['tau2']:.3f}"
    )
    print("Subgroups (descriptive)")
    for g, s in res["subgroups"].items():
        print(f"  {g}: k = {s['k']}, N = {s['N']}, g = {ci(s)}, I2 = {s['I2']:.1f}%")
    t = res["subgroup_test"]
    print(f"  Between-subgroup Q = {t['Qb']:.2f}, p = {t['p']:.2f}")
    print("Sensitivity analyses (Table S7)")
    for l in res["loo"]:
        print(f"  Omitting {l['omitted']}: g = {ci(l)}, I2 = {l['I2']:.1f}%")
    print(f"  Adding Erdem in-situ arm: g = {ci(res['add_erdem_insitu'])}")
    print(f"  Adding Erdem arms combined: g = {ci(res['add_erdem_combined'])}")
```

Table S10. PRISMA 2020 checklist (Page et al., BMJ 2021;372:n71), completed for the revised manuscript. Locations refer to section, table, and figure numbers in the revised manuscript and this file.

Section and topic	Item	Checklist item	Location where item is reported
**TITLE**			
Title	1	Identify the report as a systematic review.	Title
**ABSTRACT**			
Abstract	2	See the PRISMA 2020 for Abstracts checklist.	Abstract (structured: background/objectives, methods, results, conclusions)
**INTRODUCTION**			
Rationale	3	Describe the rationale for the review in the context of existing knowledge.	Section 1, paragraphs 1–4
Objectives	4	Provide an explicit statement of the objective(s) or question(s) the review addresses.	Section 1, final paragraph
**METHODS**			
Eligibility criteria	5	Specify the inclusion and exclusion criteria for the review and how studies were grouped for the syntheses.	Sections 2.2 and 2.3; operational definition of TEL in Section 1 and 2.2
Information sources	6	Specify all databases, registers, websites, organisations, reference lists and other sources searched or consulted to identify studies. Specify the date when each source was last searched or consulted.	Section 2.4; Table S1 (MEDLINE via PubMed, Scopus, CINAHL, CENTRAL; final searches 7 October 2026)
Search strategy	7	Present the full search strategies for all databases, registers and websites, including any filters and limits used.	Table S1 (verbatim strategies, limits, record counts); Figure S4 (search-history records)
Selection process	8	Specify the methods used to decide whether a study met the inclusion criteria of the review, including how many reviewers screened each record and each report retrieved, whether they worked independently, and if applicable, details of automation tools used in the process.	Section 2.5 (two independent reviewers; third reviewer for disagreements; no automation tools)
Data collection process	9	Specify the methods used to collect data from reports, including how many reviewers collected data from each report, whether they worked independently, any processes for obtaining or confirming data from study investigators, and if applicable, details of automation tools used in the process.	Section 2.6 (piloted form; two reviewers; source page and table recorded for each value; rule for discrepant values)
Data items	10a	List and define all outcomes for which data were sought. Specify whether all results that were compatible with each outcome domain in each study were sought (e.g. for all measures, time points, analyses), and if not, the methods used to decide which results to collect.	Section 2.2 (Outcomes); Section 2.3 (definition of knowledge vs classification outcomes); Section 2.6 (first post-intervention time point; one knowledge outcome per study)
	10b	List and define all other variables for which data were sought (e.g. participant and intervention characteristics, funding sources). Describe any assumptions made about any missing or unclear information.	Section 2.6; File S2 (all extracted variables). Missing values were not imputed. Funding sources of included studies were not extracted.
Study risk of bias assessment	11	Specify the methods used to assess risk of bias in the included studies, including details of the tool(s) used, how many reviewers assessed each study and whether they worked independently, and if applicable, details of automation tools used in the process.	Section 2.7 (RoB 2, ROBINS-I, NHLBI before–after tool; two independent reviewers)
Effect measures	12	For each outcome, specify the effect measure(s) (e.g. risk ratio, mean difference) used in the synthesis or presentation of results.	Section 2.8 (Hedges’ g with small-sample correction)

Section and topic	Item	Checklist item	Location where item is reported
Synthesis methods	13a	Describe the processes used to decide which studies were eligible for each synthesis (e.g. tabulating the study intervention characteristics and comparing against the planned groups for each synthesis (item #5)).	Section 2.3; Table 1 (synthesis assignment and reason for each study)
	13b	Describe any methods required to prepare the data for presentation or synthesis, such as handling of missing summary statistics, or data conversions.	Section 2.6; Section 2.8 (Special designs: matched-pairs study; three-month assessment; combining arms of a multi-arm trial)
	13c	Describe any methods used to tabulate or visually display results of individual studies and syntheses.	Section 2.8; Table 1; Figure 2 (forest plot); Tables S2 and S6
	13d	Describe any methods used to synthesize results and provide a rationale for the choice(s). If meta-analysis was performed, describe the model(s), method(s) to identify the presence and extent of statistical heterogeneity, and software package(s) used.	Section 2.8 (DerSimonian–Laird random effects, Wald CIs; Q, I^2, τ^2, prediction interval; Python 3.13 with NumPy and SciPy, cross-checked with statsmodels 0.15 and a spreadsheet; Code S1)
	13e	Describe any methods used to explore possible causes of heterogeneity among study results (e.g. subgroup analysis, meta-regression).	Section 2.8 (prespecified subgroup: nursing students vs registered nurses; descriptive because one subgroup has a single study)
	13f	Describe any sensitivity analyses conducted to assess robustness of the synthesized results.	Section 2.8 (leave-one-out; HKSJ; Paule–Mandel; common-effect model; addition of the excluded physical-simulation trial, by single arm and with arms combined)
Reporting bias assessment	14	Describe any methods used to assess risk of bias due to missing results in a synthesis (arising from reporting biases).	Section 2.8 (Small-study effects: funnel plot; formal tests not performed with fewer than ten studies)
Certainty assessment	15	Describe any methods used to assess certainty (or confidence) in the body of evidence for an outcome.	Section 2.9 (GRADE; starting certainty for a body with ROBINS-I; optimal information size assumptions; indirectness criteria); Table S9
**RESULTS**			
Study selection	16a	Describe the results of the search and selection process, from the number of records identified in the search to the number of studies included in the review, ideally using a flow diagram.	Section 3.1; Figure 1 (4,195 identified; 2,146 duplicates; 2,049 screened; 175 assessed; 170 excluded; 5 included, 3 in meta-analysis)
	16b	Cite studies that might appear to meet the inclusion criteria, but which were excluded, and explain why they were excluded.	Section 3.1; Table S8
Study characteristics	17	Cite each included study and present its characteristics.	Section 3.2; Table 1
Risk of bias in studies	18	Present assessments of risk of bias for each included study.	Section 3.3; Tables S3–S5 (domain-level judgments and rationale); Figures S1–S2
Results of individual studies	19	For all outcomes, present, for each study: (a) summary statistics for each group (where appropriate) and (b) an effect estimate and its precision (e.g. confidence/credible interval), ideally using structured tables or plots.	Sections 3.4 and 3.8; Figure 2; Table S2 (group means, SDs, sample sizes); Table S6 (effect sizes, variances, SEs, CIs, weights)
Results of syntheses	20a	For each synthesis, briefly summarise the characteristics and risk of bias among contributing studies.	Sections 3.2–3.4
	20b	Present results of all statistical syntheses conducted. If meta-analysis was done, present for each the summary estimate and its precision (e.g. confidence/credible interval) and measures of statistical heterogeneity. If comparing groups, describe the direction of the	Section 3.4; Figure 2 ($g = 1.33$, 95% CI 0.98 to 1.69; $I^2 = 41.5\%$; $\tau^2 = 0.041$; favours TEL)

Section and topic	Item	Checklist item	Location where item is reported
		effect.	
	20c	Present results of all investigations of possible causes of heterogeneity among study results.	Section 3.5; Figure 2
	20d	Present results of all sensitivity analyses conducted to assess the robustness of the synthesized results.	Section 3.6; Table 2; Table S7; Figure S3
Reporting biases	21	Present assessments of risk of bias due to missing results (arising from reporting biases) for each synthesis assessed.	Section 3.7; Figure 3
Certainty of evidence	22	Present assessments of certainty (or confidence) in the body of evidence for each outcome assessed.	Section 3.9; Table 3; Table S9 (low certainty)
**DISCUSSION**			
Discussion	23a	Provide a general interpretation of the results in the context of other evidence.	Sections 4.1 and 4.2
	23b	Discuss any limitations of the evidence included in the review.	Section 4.4
	23c	Discuss any limitations of the review processes used.	Section 4.4
	23d	Discuss implications of the results for practice, policy, and future research.	Sections 4.3 and 4.5
**OTHER INFORMATION**			
Registration and protocol	24a	Provide registration information for the review, including register name and registration number, or state that the review was not registered.	Section 2.1 (not registered)
	24b	Indicate where the review protocol can be accessed, or state that a protocol was not prepared.	Section 2.1 (no protocol was published)
	24c	Describe and explain any amendments to information provided at registration or in the protocol.	Sections 2.1 and 2.5 (changes made during peer review identified)
Support	25	Describe sources of financial or non-financial support for the review, and the role of the funders or sponsors in the review.	Funding statement (no specific funding)
Competing interests	26	Declare any competing interests of review authors.	Conflicts of Interest statement (none)
Availability of data, code and other materials	27	Report which of the following are publicly available and where they can be found: template data collection forms; data extracted from included studies; data used for all analyses; analytic code; any other materials used in the review.	Data Availability Statement; File S2 (extraction matrix, which shows all data-collection fields, and analysis data); Tables S2 and S6; Code S1 and File S3 (analytic code)
